# Recombinant human PRG4 (rhPRG4) suppresses breast cancer cell invasion by inhibiting TGFβ-Hyaluronan-CD44 signalling pathway

**DOI:** 10.1371/journal.pone.0219697

**Published:** 2019-07-30

**Authors:** Anusi Sarkar, Ayan Chanda, Suresh C. Regmi, Kunal Karve, Lili Deng, Gregory D. Jay, Frank R. Jirik, Tannin A. Schmidt, Shirin Bonni

**Affiliations:** 1 The Arnie Charbonneau Cancer Institute and Department of Biochemistry & Molecular Biology, The Cumming School of Medicine, University of Calgary, Calgary, Alberta, Canada; 2 Faculty of Kinesiology, University of Calgary, Calgary, Alberta, Canada; 3 Department of Emergency Medicine—Alpert Medical School & School of Engineering, Brown University, Providence, Rhode Island, United States of America; 4 Biomedical Engineering Department, University of Connecticut Health Center, Farmington, Connecticut, United States of America; Beijing Cancer Hospital, CHINA

## Abstract

Metastasis is the major cause of cancer-related morbidity and mortality. The ability of cancer cells to become invasive and migratory contribute significantly to metastatic growth, which necessitates the identification of novel anti-migratory and anti-invasive therapeutic approaches. Proteoglycan 4 (PRG4), a mucin-like glycoprotein, contributes to joint synovial homeostasis through its friction-reducing and anti-adhesive properties. Adhesion to surrounding extracellular matrix (ECM) components is critical for cancer cells to invade the ECM and eventually become metastatic, raising the question whether PRG4 has an anti-invasive effect on cancer cells. Here, we report that a full-length recombinant human PRG4 (rhPRG4) suppresses the ability of the secreted protein transforming growth factor beta (TGFβ) to induce phenotypic disruption of three-dimensional human breast cancer cell-derived organoids by reducing ligand-induced cell invasion. In mechanistic studies, we find that rhPRG4 suppresses TGFβ-induced invasiveness of cancer cells by inhibiting the downstream hyaluronan (HA)-cell surface cluster of differentiation 44 (CD44) signalling axis. Furthermore, we find that rhPRG4 represses TGFβ-dependent increase in the protein abundance of CD44 and of the enzyme HAS2, which is involved in HA biosynthesis. It is widely accepted that TGFβ has both tumor suppressing and tumor promoting roles in cancer. The novel finding that rhPRG4 opposes HAS2 and CD44 induction by TGFβ has implications for downregulating the tumor promoting roles, while maintaining the tumor suppressive aspects of TGFβ actions. Finally, these findings point to rhPRG4’s potential clinical utility as a therapeutic treatment for invasive and metastatic breast cancer.

## Introduction

Cancer is caused by uncontrolled proliferation and increased survival of cells leading to primary tumor formation, followed by a population of the primary tumor cells acquiring the ability to invade and spread within the primary tumor vicinity or to distant sites, where they can form secondary tumors or metastases [1]. In breast cancer, which is the most frequently detected cancer in women worldwide, metastasis is responsible for 90% of breast cancer-related deaths [2].

Development of novel targeted therapies to combat various types of cancers has been enabled by the discovery of altered membrane or intracellular components in cancer cells as compared to normal cells [3]. In the case of breast cancer, therapeutics that bind and inhibit the function of estrogen receptor (ER), progesterone receptor (PR), and human epidermal growth factor receptor 2 (HER2) have led to enhanced survival rates of patients whose tumors are characterized as ER positive, PR positive, and HER2 amplified, respectively [4]. Breast tumors that are ER and PR negative, with no HER2 amplification, are termed as triple negative breast cancer (TNBC). TNBCs account for only 10 to 20% of all diagnosed breast cancer cases, but are highly metastatic and associated with poor prognosis and worst mortality rates as compared to other breast cancer molecular subtypes [5]. The treatments of TNBCs are limited mainly to conventional chemotherapeutics, e.g. taxanes or platinum-based agents, which are associated with significant adverse effects, resistance development and tumor recurrence [6]. Thus, novel therapeutics that can target the TNBC subtype with diminished or no off-target toxicity are urgently needed in cancer management.

Proteoglycan 4 (PRG4, also known as lubricin) is a mucin-like glycoprotein originally discovered in synovial fluid as a secreted product of cells lining joint tissues, which is also present at the surface of articular cartilage [7]. PRG4 is classically described as a boundary lubricant, present in synovial fluid at ~100–500 μg/mL concentration, that reduces the friction between articulating cartilage surfaces during movement thus contributing to joint integrity [8]. However, recently PRG4 has also been demonstrated to have anti-inflammatory properties [9–13]. PRG4 has cysteine rich globular N and C termini that facilitate both intra and inter molecular disulfide bonding, and contain somatomedin B and hemopexin like domains, respectively. PRG4 also contains a central mucin-like domain, which is post-translationally modified with β (1–3) galactose (Gal) and N-Acetyl-D-galactosamine (GalNAc), with an incomplete capping of sialic acid [7]. This extensive O-linked glycosylated mucin-like domain is necessary for PRG4’s boundary lubricating and dis-adhesive properties at various biointerfaces in the body including articular cartilage, tendons, the pericardium, and the ocular surface [7].

Recently, full-length recombinant human PRG4 (rhPRG4) protein has been expressed successfully at large scale making it available for basic and translational-based investigations. rhPRG4 has been shown to retain appropriate higher order structure and glycosylations, and thus displays efficient *in vitro* lubricating and anti-adhesive functions [14,15]. Importantly, rhPRG4 provides effective *in vivo* therapeutic value in preservation of joint health via intra-articular injection in preclinical *in vivo* osteoarthritis models [16,17]. More importantly, rhPRG4 was shown to be effective in a small clinical trial (NCT02507934) resulting in improving signs and symptoms of patients with dry eye disease [18]. While the mechanism of action remains to be determined, be it mechanical (lubrication and anti-adhesive) [8] and/or biological [10], this study demonstrates the clinical utility of rhPRG4 [18]. A key question from these findings is whether rhPRG4 can be used in other clinical settings including cancer.

The focus of the current study is to define the role of rhPRG4 in tumor cell responses. The abilities of tumor cells to become migratory and invasive contribute significantly to their metastatic potential. Cancer cells' interactions with the ECM can promote cell migration and invasion. Given its dis-adhesive properties, rhPRG4 effect on tumor cell invasion and migration were investigated [19,20]. The findings reported in this study suggest the exciting idea that rhPRG4 suppresses TNBC breast cancer cell migration and invasiveness. These findings point to rhPRG4 as a potential novel cancer therapeutic.

## Materials and methods

### Plasmids

The pU6/CD44 RNA interference-1/CMV-enhanced green fluorescent protein (EGFP) and pU6/CD44 RNA interference-2/CMV-EGFP expression vectors, abbreviated as CD44i-1 and CD44i-2, containing the CD44 sequences 5’GAGCAGCACTTCAGGAGGTTA3’ and 5’CTCCATCTGTGCAGCAAACAA3’, respectively, were generated as described [21]. In each of the plasmids CD44i-1 and CD44i-2, the mouse U6 small RNA promoter and CMV promoter induce the expression, respectively, of a human CD44 mRNA-targeting short hairpin (sh) RNAs (shRNAs) and EGFP. The pU6/CMV/EGFP control RNAi vector has been described [21]. Expression of the EGFP protein, visualized by fluorescence microscopy, indicated vector control or CD44i-1/2 transfected cells. The pCMV5B/CD44/FLAG expression vector was generated by a T4 DNA ligase (New England BioLabs, USA)-based ligation of a C-terminally FLAG-tagged human open reading CD44 cDNA (CD44/FLAG) into the pCMV5B vector [22]. CD44/FLAG DNA was generated by a polymerase chain reaction (PCR) using Pwo polymerase (Roche Diagnostics, USA), polyA-enriched cDNA as template, and 5’ CCCACGCGTACCATGGACAAGTTTTGGTGGC 3’, and 5’ CCCTCTAGATTACTTGTCATCGTCGTCCTTGTAGTCCAGTCGACCCACCCCAATCTTCATGTCC 3', respectively, as forward and reverse primers. The poly A cDNA was generated by subjecting MDA-MB-231 cell TRIzol-(Ambion Life Technologies, Canada) extracted mRNA to reverse transcription (RT)-PCR reaction using the SuperScript II transcriptase (Invitrogen, Canada) and the primer oligo-(dT)12-18 (Amersham Biosciences, UK). CD44i-1/2, and CD44/FLAG plasmids were verified by DNA sequence analyses (University of Calgary Core Sequencing Facility).

### Cell lines and transfections

The triple negative breast cancer (TNBC) MDA-MB-231 cells, obtained from American Type Culture Collection (ATCC, USA), were cultured in Dulbecco’s modified Eagle medium (DMEM; Invitrogen, Canada) supplemented with 10% fetal bovine serum (FBS; Thermo Fisher, Canada). The TNBC HCC38 cells (ATCC), a generous gift from Dr. Don Morris’s laboratory (University of Calgary), were cultured in Roswell Park Memorial Institute medium 1640 (RPMI 1640; Life Technologies, Canada) supplemented with 10% FBS (Thermo Fisher, Canada). MDA-MB-231 and HCC38 cells were kept at 37°C in a 5% CO_2_ humidified cell incubator, and were routinely passaged every 3–4 days, and were transfected using Lipofectamine 3000 reagents (Invitrogen, Canada).

### Reagents

The following reagents were used in the study: recombinant human proteoglycan 4 (rhPRG4; Gift from Lμbris Biopharma, USA; stock 1 to 2.5 mg/mL in phosphate-buffered saline (PBS)) [14,15], recombinant human mature transforming growth factor beta (TGFβ) (R&D systems, USA; stock 10μM), Low molecular weight hyaluronic acid (LMWHA; Sodium Hyaluronate; HA5K; Lifecore Biomedical, USA; stock 10 mg/mL in PBS), Kinase inhibitor (KI; SB431542; Millipore-Sigma, Canada; stock 10 mM) [23], CD44 neutralizing antibody (Thermo Fisher Scientific, Canada) [24], Anti-PRG4 mAb 4D6 (Gift from Dr. Phillip Messersmith, University of California Berkeley) [25,26], Mouse Immunoglobulin G (Mouse IgG; Santa Cruz, USA), 4-methylumbelliferone (4-MU; Millipore-Sigma, Canada; stock 10 mM in DMSO).

### Three-dimensional cultures

For three-dimensional culture assays, 50 μL of ice-cooled 33% growth factor-reduced Matrigel (Corning Incorporated, USA) in complete growth medium, consisting of DMEM containing 10% FBS, penicillin, streptomycin and amphotericin B (Invitrogen, Canada), was added per well of a 96-well flat bottom, ultra-low attachment plate (BD Biosciences, Canada) followed by storing the plate for 1h in a 5% CO_2_ humidified incubator at 37°C to allow formation of a 1 mm-thick Matrigel bed in the well. Next, approximately 400 MDA-MB-231 or 800 HCC38 isolated cells were suspended in 50 μL ice-cooled 50% Matrigel in complete growth medium and layered on top of the Matrigel bed per well of the 96-well plate and incubated inside 5% CO_2_ humidified incubator at 37°C. 1h post addition and 37°C incubation of Matrigel-cells suspension, 50 μL of complete growth medium was added to cover the solidified Matrigel-cells mixture. The following day and, every third day, the three-dimensional cultures received 50 μL of fresh complete growth medium without or with specified reagents as listed in the figure legends. (Note that 100pM of TGFβ is considered to be close to saturating concentration given the high binding affinity of the ligand for the TGFβ receptor I and II, namely the equilibrium dissociation constant (Kd) is approximately 50pM [27]). Observation of the Matrigel-embedded cultures by light microscopy at different days of three-dimensional cultures, indicated that isolated cells, after undergoing successive divisions, each gave rise to a multicellular structure, here referred to as an organoid. 8-day old 3D-MDA-MB-231 or 6-day old HCC38 cell-derived organoids were inspected using differential interference contrast (DIC) light microscopy with images of 8 representative multicellular structures captured from each well at 30X objective (Olympus IX70, Canada). In the control setting, the majority of the MDA-MB-231 and HCC38-cell-derived organoids displayed smooth-surfaced spherical phenotype, which can change depending on the incubation conditions of the 3D cultures. The number of smooth-surfaced organoids without any protrusions, designated as “spherical” were expressed as a percentage of the total eight representative organoids whose images were captured in each well. Each experiment was repeated at least three independent times and percentage spherical organoids were subjected to ANOVA statistical analyses, and are presented as mean ± SEM of the independent replicates on the y-axis versus experimental condition on the x-axis of a bar graph.

### Immunocytochemistry and fluorescence-cell based analyses

For immunofluorescence analysis of 3D-organoids, 600 MDA-MB-231 or 1000 HCC38 isolated cells were cultured within 80 μL of 50% Matrigel on top of 80 μL of 33% Matrigel bed in each well of ultra-low attachment 8-well chamber slides (Millipore-Sigma, USA). After DIC inspection and imaging, the live multicellular structures in the 3D-cultures were fixed with 4% formaldehyde, followed by permeabilization using 0.5% ice-cold Triton X-100 solution and blocking using 10% bovine serum albumin (BSA) in phosphate-buffered saline (PBS). For immunofluorescence analysis of monolayer cell culture, cells were seeded onto wells of a Falcon 8-well chamber culture slides (Corning Incorporated, USA), followed by fixing and permeabilization as described for the 3D cultures, and blocking with 5% BSA and 5% calf serum in PBS. The proteins laminin and CD44 in cells were visualized by subjecting the three-dimensional or monolayer cultures to indirect immunofluorescence using a rat anti-laminin antibody (Abcam, Canada) and rat anti-CD44 antibody (Thermo Fisher, Canada) as the primary antibody, respectively, and Alexa 647-conjugated anti-rat IgG (ThermoFisher, Canada) as the secondary antibody. Actin in cells was visualized by incubating the fixed cultures with tetramethylrhodamine isothiocyanate (TRITC)-conjugated phalloidin (Millipore-Sigma, Canada). The DNA binding dye bisbenzimide (Hoechst 33342; Invitrogen, Canada) was used to detect nuclei. For CD44 knockdown analysis, the vector or CD44 shRNA transfected cells were identified by GFP signal. Immunofluorescence images were captured using an epifluorescence microscope with a 40X objective lens (Olympus Bx WI Confocal Microscope, Canada). Exposure times for each of laminin, actin, nuclei and GFP-specific signals were kept constant in each experiment. For each condition, 3 colonies/field per experiment were captured, which were chosen as representatives of the stained cells within each slide per experiments. Each experiment was repeated two independent times.

### *In vitro* transwell invasion assays

Overnight 0.2% FBS-containing DMEM incubated, i.e. serum-starved, MDA-MB-231 cells were used for the transwell invasion using polycarbonate filters (24-well inserts, pore size 8 μm; BD Biosciences, Canada). Prior to addition of cells, each insert was placed within a well of a 24-well tissue culture plate and equilibrated with 0.5 mL serum-free DMEM, added both to the upper and lower chambers at 37°C for 2h. The equilibration media was then gently removed and upper chamber surface of the insert was coated with 50 μL of 3% Matrigel and allowed to solidify at 37°C for 1h. 1X10^5^ serum-starved MDA-MB-231 cells were resuspended in 0.5 mL of serum-free DMEM and added to the upper Matrigel-coated chamber. 500 μL complete growth medium alone or containing specific reagents, as described in the respective figure legends, was added to the lower chamber. Cells were allowed to invade the matrix for 12h at 37°C after which non-adherent cells were removed by PBS washing of cell layers on the upper chamber three times. During the second wash a cotton tip applicator was used to gently scrape away the adherent cells on the upper surface of the membrane. Invading cells were fixed by immersing the transwell inserts in 100% methanol for 10 minutes at -20°C, followed by staining with 0.5% crystal violet dye (EMD Millipore, Canada) for 1h at room temperature. Eight randomly chosen fields of each stained membrane were imaged at 10X objective of a DIC microscope (Olympus IX70) coupled to a digital camera. Crystal violet-stained cells in each field were counted using a handheld counter and an average count of cells for the 8 fields per condition was obtained. Each experiment was repeated at least three independent times, and invading cell counts at each experimental condition were expressed relative to an experimental global average and subjected to statistical analysis. The mean ± SEM of relative invading cells of the independent experiments is plotted on the y-axis versus the experimental conditions on the x-axis of a bar graph.

### *In vitro* scratch assays

5×10^5^ MDA-MB-231 cells were seeded in each well of a 12-well tissue culture plate and grown for 24h to near confluency in complete growth medium and then 24h serum starved by incubating with 0.2% FBS-containing DMEM medium in a 5% CO2 humidified incubator at 37°C incubator. Using a 200μL pipette tip, a scratch was introduced along the midline of the serum-starved cell monolayers, followed by a PBS wash to remove floating cells, and incubating the cells with 0.2% FBS-containing medium without or with TGFβ, alone or together with KI or rhPRG4 for 36h in a 5% CO2 humidified incubator at 37°C. Scratch closure in each well was followed by imaging the scratch and surrounding cells in each well at 3X objective of a DIC microscope (Olympus IX70) coupled to a digital camera at time 0h and 36h after initiating the scratch. Five images were captured along the vertical axis of the scratch for each experimental condition. The width of each scratch was measured at three different positions per image for a total of 15 measurements using ImageJ (National Institutes of Health, USA), and then averaged per experimental condition. The width average at 36h was subtracted from the width average at 0h and expressed relative to that at 0h width for each experimental condition to obtain scratch closure, and expressed as percent scratch closure.

### Cell extract preparation, immunoprecipitation and immunoblotting

Cells were washed with PBS to remove all growth media. Appropriate volume of TNTE lysis buffer (50 mM Tris, 150 mM NaCl, 1 mM EDTA, 0.5% [v/v] Triton-X-100) containing protease and phosphatase inhibitors, was added to the cell monolayer and incubated at 4°C under vigorous shaking for 20 minutes. Lysates were then collected in microcentrifuge tubes and centrifuged at 13,000g for 10 minutes at 4°C. 5 μL of each lysate was subjected to protein concentration determination using Bradford-based protein assays (Bio-Rad Laboratories, Canada). Lysates were boiled for 3 minutes at 95°C in dithiothreitol (DTT)-containing Laemmli sample buffer. For immunoprecipitation analyses, lysates were incubated with appropriate antibodies at 4°C with gentle rocking for 3h after which immunocomplexes were incubated with Protein G-conjugated agarose beads (UBPBio, USA) at 4°C with gentle rocking for 3h. Finally, the beads were washed with TNTE wash buffer (0.1% [v/v] Triton-X-100) and boiled for 5 minutes at 95°C in DTT containing Laemmli sample buffer. The proteins in the immunoprecipitates and input lysates were then resolved by sodium dodecyl sulfate-polyacrylamide electrophoresis (SDS-PAGE) and transferred onto a nitrocellulose membrane (Bio-Rad Laboratories, Canada). The blots were blocked using 5% skim milk followed by overnight incubation with mouse anti-actin (Santa Cruz, USA), rabbit anti-pSmad2 (Abcam, Canada), mouse anti-Smad2/3 (Millipore-Sigma, Canada), rat anti-CD44 (Thermo Fisher, Canada), mouse anti-CD44 (Santa Cruz, USA), mouse anti-HAS2 (Santa Cruz, USA) or mouse anti-FLAG (Millipore-Sigma, Canada) as the primary antibody at 4°C. Then HRP-conjugated goat anti-mouse or anti-rabbit IgG (Jackson Laboratories, USA) or anti-rat IgG (Millipore-Sigma, Canada) was added to the blots for 1h at room temperature, followed by enhanced chemiluminescence (Millipore-Sigma, Canada) and signal detection using a VersaDoc 5000 Imager (Bio-Rad Laboratories). Densitometric analyses were performed using Quantity One software (Bio-Rad Laboratories, Canada).

### Reporter assays

MDA-MB-231 cells were seeded in 24-well plates at approximately 6X10^4^ cells per well one day prior to transfections. Cells were co-transfected with the PAI1-promoter-driven firefly luciferase reporter (3TP-Lux) and the CMV-Renilla luciferase control reporter constructs. 18h post transfection, cells were serum-starved (0.2% FBS containing DMEM) for 4h and then incubated in fresh low-serum (0.2% FBS) containing DMEM medium in the absence or presence of 100 pM TGFβ, 100 μg/mL rhPRG4 alone or together and left overnight. Lysates were prepared and analyzed for luciferase activity using a commercially available dual luciferase assay kit (Promega, Canada). Arbitrary luciferase activity (relative light units) values were normalized to Renilla luciferase activity to account for variations in transfection efficiency. For each transfection, increase of PAI1 promoter driven luciferase reporter gene expression was also determined and expressed relative to luciferase activity of the respective basal condition lysates. Each experimental condition was carried out in triplicate.

### Statistical analysis

Biochemical and organoid-related data were subjected to statistical analysis by Student's t-test or One-way analysis of variance (ANOVA) followed by Tukey-Kramer or Student-Newman-Keuls post hoc test using InStat (Graphpad, USA). Values of P<0.05 were considered statistically significant. Data were presented graphically as mean ± SEM from experiments that were repeated at least three independent times.

## Results

### rhPRG4 promotes anti-invasive growth of 3D-breast cancer cell-derived organoids

To evaluate the effect of rhPRG4 on the invasiveness of breast cancer cells, we employed a three-dimensional (3D) cell culture system where cells are allowed to grow and interact with ECM components, such as Matrigel which resembles the ECM that is found *in vivo* [28]. Cellular responses to stimuli like growth factors, hormones or drugs in three dimensional culture models, as opposed to monolayer models, have been reported to better predict the response observed in a living organism and is therefore more appropriate to model the growth and assess the response to external stimuli by normal and tumor cells [29].

**Fig 1:**
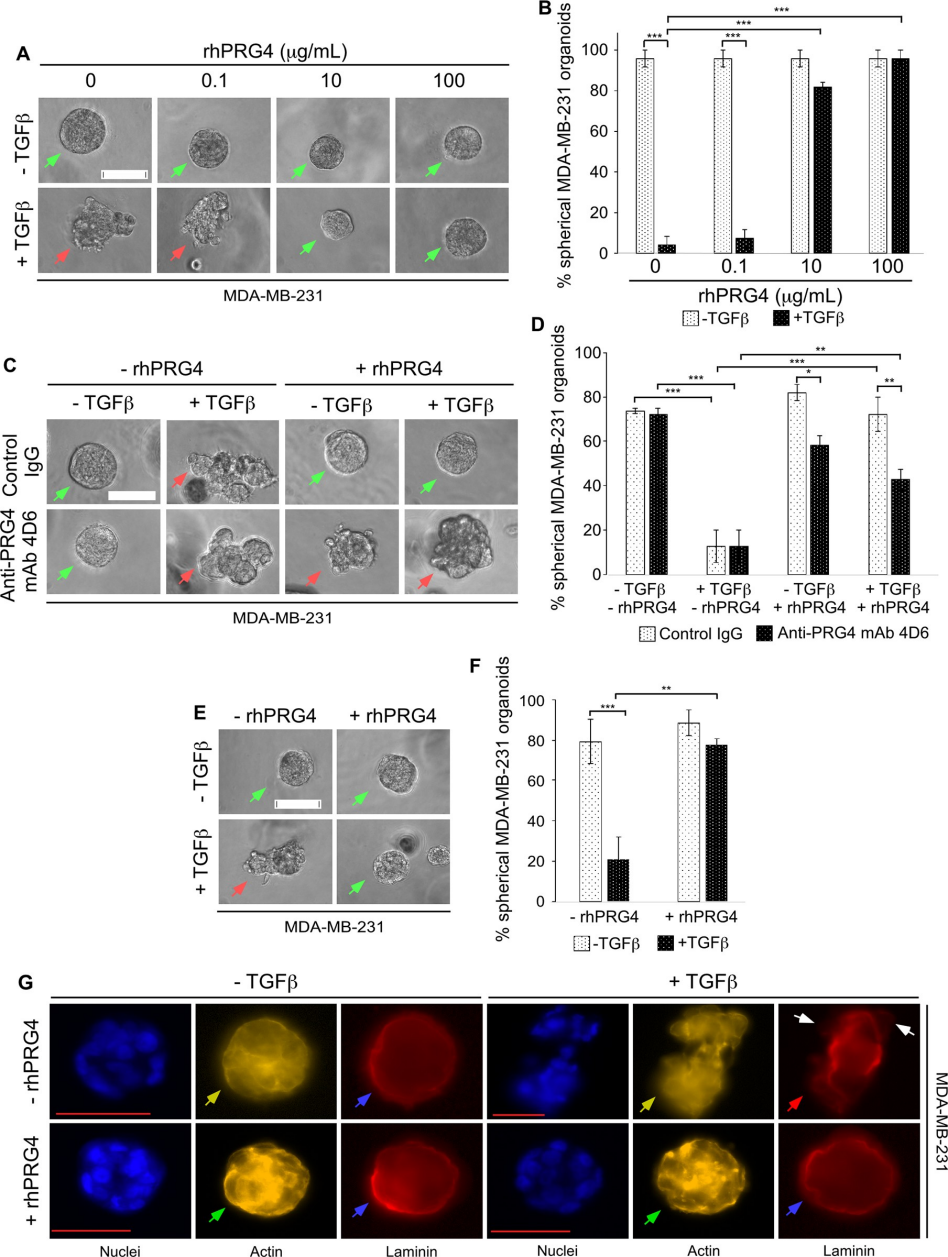
rhPRG4 suppresses TGFβ-induced invasive growth of MDA-MB-231 cell-derived organoids. **(A)** Representative DIC light microscopy images of 8-day old three-dimensional MDA-MB-231 cell-derived organoids that were left untreated or incubated with 100 pM TGFβ, without or with increasing concentrations of rhPRG4 (0.1, 10 and 100 μg/mL) in complete growth medium. **(B)** Bar graph depicts mean ± SEM proportion of spherical organoids expressed as a percentage of total colonies counted for each experimental condition from four independent experiments including the one shown in A. **(C)** Representative DIC light microscopy images of 8-day old three-dimensional MDA-MB-231 cell-derived organoids that were incubated with 10 μg/mL of mouse IgG or anti-PRG4 mAb 4D6, along with or without 100 pM TGFβ and 100 μg/mL rhPRG4 in different combinations in complete growth medium. **(D)** Bar graph depicts mean ± SEM proportion of spherical organoids expressed as a percentage of total colonies counted for each experimental condition from three independent experiments including the one shown in C. **(E)** Representative DIC light microscopy images of untreated or 100 pM TGFβ-treated 8-day old three-dimensional MDA-MB-231 cell-derived organoids using Matrigel that was mixed with vehicle or 100 μg/mL rhPRG4. **(F)** Bar graph depicts mean ± SEM proportion of spherical organoids expressed as a percentage of total colonies counted for each experimental condition from three independent experiments including the one shown in E. **(G)** Representative fluorescence microscopy images of nuclear (Hoechst 33342, blue), actin (TRITC- phalloidin, yellow), and laminin (rat anti-laminin/anti-rat Alexa 647, red) staining of formaldehyde-fixed 8 day old MDA-MB-231 cell-derived organoids that were left untreated or incubated with TGFβ, with or without rhPRG4, in complete growth medium. This experiment was repeated two independent times with similar outcomes. Significant difference, ANOVA: *P ≤ 0.05, **P ≤ 0.01, ***P ≤ 0.001. Scale bar indicates 50 μm. For Fig 1A, 1C and 1E, green and red arrows indicate spherical and invasive organoids, respectively. For Fig 1G, green arrows indicate cortical actin, yellow arrows indicate stress-fibre like actin, blue arrows indicate intact laminin rings, red arrow indicates disruption of laminin ring, and white arrows indicate representative sites of laminin loss.

To study the potential anti-invasive effect of rhPRG4 on TNBC-derived cells, we employed a three-dimensional model, where the migratory and invasive behaviour of these tumor cells in response to certain stimuli can be easily detected and quantified. The human TNBC MDA-MB-231 breast cancer cell line represents a widely used TNBC cell model for *in vitro* and *in vivo* cancer studies including in three-dimensional culture models [30,31], thus we used these breast cancer cells in our investigations. As expected, we found that isolated single MDA-MB-231 cells cultured in the context of Matrigel, as an extracellular support system, divided and formed multicellular aggregates that mostly displayed smooth-surfaced spherical phenotypes (**Fig 1A and 1B**). The secreted polypeptide transforming growth factor β (TGFβ) plays a complex role in cancer [32]. In particular, TGFβ can promote migration and invasion, and may thus contribute to cancer metastasis [32]. The ability of TGFβ to remodel ECM components of the tumor microenvironment contributes to its role in increasing migration and invasion of tumor cells [32]. As predicted, we found that TGFβ disrupted the smooth surface and spherical nature of 3D-Matrigel-MDA-MB-231 cell-derived organoids, and promoted a disruptive phenotype of these organoids [30,31]. rhPRG4 acted in a dose-dependent manner to repress TGFβ-induced disruption of 3D-breast cancer cell-derived organoids. The anti-PRG4 monoclonal antibody (mAb) 4D6 specifically recognizes PRG4 [25,26]. We found that incubation of the 3D cultures with the anti-PRG4 mAb 4D6, but not with mouse IgG control, decreased the ability of rhPRG4 to suppress TGFβ-induced deformation in the growth of the 3D-breast cancer cell-derived organoids **(Fig 1C and 1D)**. Together, these data suggested that rhPRG4 acts in a dose-dependent and specific manner to suppress the disruptive behaviour of MDA-MB-231 cell-derived organoids.

The findings that PRG4 acts in a specific manner to preserve the spherical phenotype of the TNBC-derived organoids even in the presence of TGFβ, raised the key question whether addition of PRG4 to the Matrigel prior to addition of isolated cells is sufficient to counteract TGFβ-induced disruption in growth of breast cancer cell-derived organoids. Remarkably, applying rhPRG4 to the Matrigel even prior to the setting of the three-dimensional culture was sufficient to maintain the spherical nature of the 3D-TNBC derived organoids (**Fig 1E and 1F)**.

Basal lamina disruption and cortical to stress-fibre-like actin reorganization are two requisite factors for cells to become invasive [33,34]. It is important to note that the ECM protein laminin is enriched at the basal lamina of organoids maintaining their structural integrity and polarity, and this enrichment is gradually lost with increase in neoplastic tendency [28,35]. Thus, next, the effect of rhPRG4 on the ability of TGFβ to alter the basal lamina status and actin organization in 3D-MDA-MB-231-derived organoids were tested using indirect immunofluorescence analysis (laminin), fluorescently-labelled phalloidin (actin) and Hoechst (nuclei) staining. In untreated MDA-MB-231 cell-derived organoids, the basal lamina was intact as suggested by the appearance of laminin as a solid ring surrounding the outside surface of the organoid, whereas actin was partially cortically oriented **(Fig 1G)**. TGFβ disrupted basement membrane organization around the organoid as indicated by loss of the laminin ring surrounding the organoids, and promoted actin stress-fibre like appearance. These TGFβ effects are consistent with its ability to increase mobility and invasion of the cellular components of the 3D-MDA-MB-231 organoids [30,31]. rhPRG4 promoted cortical actin organization and solid laminin ring formation in these multicellular structures in the absence or presence of TGFβ. Collectively, these data suggest that TGFβ-induced disorganization and deformation of the 3D-breast cancer cell-derived organoids, at least in part, reflects an invasive response of these cells to incubation with this ligand. Moreover, our data suggest that rhPRG4 promotes an anti-invasive growth of breast cancer cells-derived organoids cultured in the context of an ECM support.

### PRG4 suppresses invasion and migration of breast cancer cells

**Fig 2:**
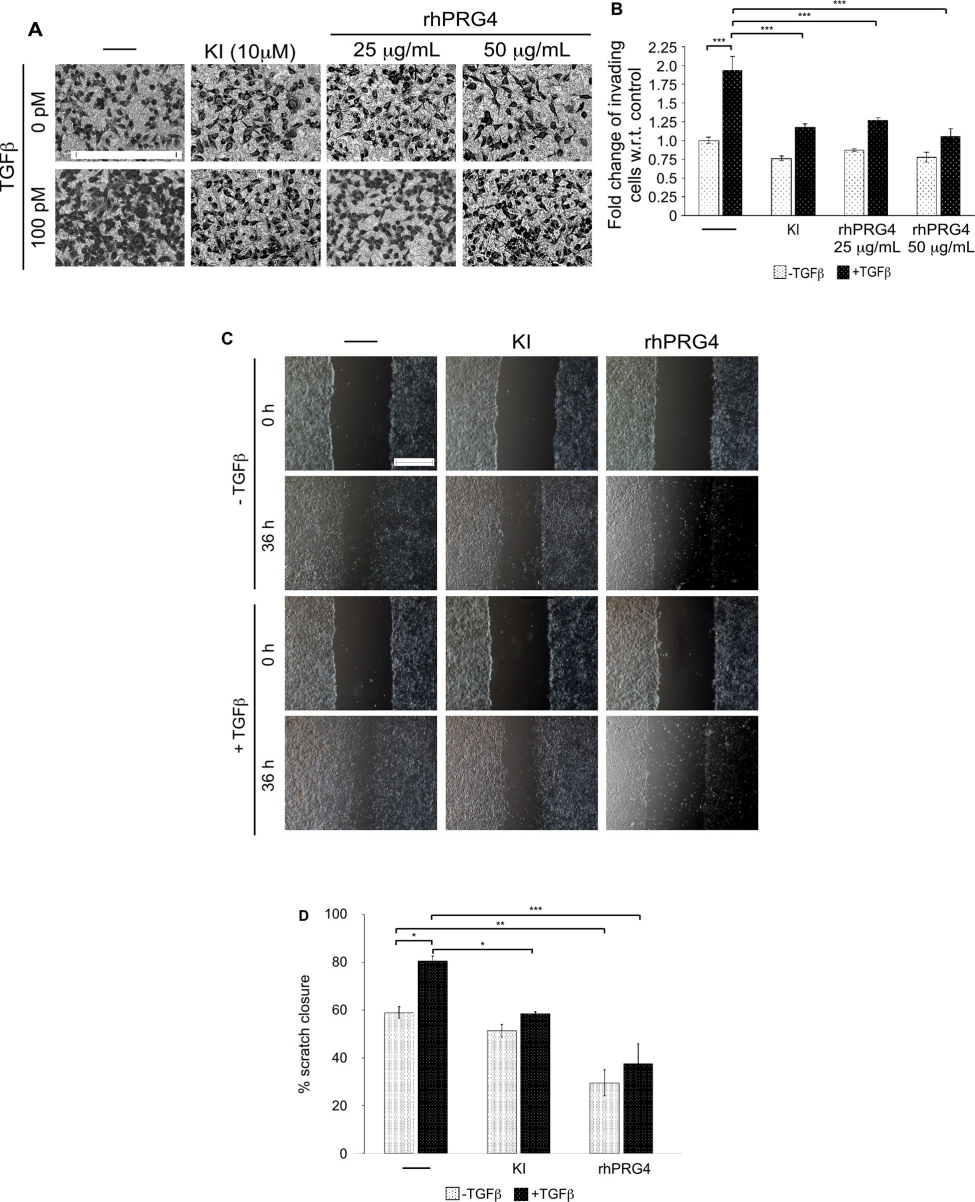
rhPRG4 suppresses breast cancer cells' invasive and migratory abilities. **(A)** Representative DIC light microscopy images of crystal violet-stained 12h-serum-starved MDA-MB-231 cells appearing on the underside of Matrigel-coated membrane of a transwell insert, with the bottom well containing complete growth medium without (-) or with 100 pM TGFβ, alone or with 10 μM of TβRI inhibitor SB435142 (KI), 25 μg/mL rhPRG4 or 50 μg/mL rhPRG4. Scale bar represents 150 μm. **(B)** Bar graph depicts mean ± SEM fold change of invaded cells relative to control counted from eight randomly chosen non-overlapping fields for each experimental conditions from five independent experiments including the one shown in A. **(C)** Representative DIC light microscopy images of serum-starved MDA-MB-231 cells seeded in wells of a 12-well plate at 0h and 36h after the introduction of a scratch, and incubated with 0.2% FBS-containing medium without (-) or with 100 pM TGFβ, alone or with 10 μM KI or 100 μg/mL rhPRG4. Scale bar represents 500μm. **(D)** Bar graph depicts mean ± SEM proportion of scratch closure (%) at 36h with respect to the 0h of 5 non-overlapping images of each experimental condition from three independent experiments including the one shown in C. Significant difference, ANOVA: *P ≤ 0.05, **P ≤ 0.01, ***P ≤ 0.001.

Our findings from the 3D-cell derived organoids suggested that rhPRG4 may favour a non-invasive growth phenotype. To further investigate this idea, the effect of rhPRG4 on cell invasion was tested using an *in vitro* transwell assay [30]. Specifically, serum-starved MDA-MB-231 cells were seeded in the upper chamber on top of a Matrigel-coated membrane, with the lower chamber having 10% FBS-containing growth medium in the absence or presence of TGFβ, alone or together with TGFβ type I ser/thr kinase receptor (TβRI) small molecule kinase inhibitor SB431542 (KI) or rhPRG4 as a chemoattractant [23]. TGFβ acted in a TβRI-signalling-dependent manner to promote the invasion of MDA-MB-231 cells as compared to untreated control (**Fig 2A and 2B**). However, rhPRG4 blocked the ability of MDA-MB-231 cells to be invasive in presence of TGFβ. In addition to invasion, migration plays an important role in the ability of cancer cells to move to sites outside the primary tumor site for metastasis [19]. Thus, *in vitro* scratch assays were performed to test the effect of PRG4 on migratory behaviour of the cancer cells. A scratch was created along the midline of 24 h-serum-starved confluent cell monolayers in each well, followed by incubation for 36h with low-serum medium without or with TGFβ, alone or with KI or rhPRG4. We, first, confirmed that during the 36h treatment period where cells are cultured at low-serum conditions, cells are quiescent at the start of the scratch and throughout the experimental observations, thus reducing the chance of cell proliferation contributing to the scratch-produced space closure (**S1 Fig**). Thus, these data support the idea that closure of the wound under low-serum conditions is primarily, if not entirely, due to cells’ migratory responses. We found that TGFβ signalling increased the scratch closure process as compared to that in the control well, whereas, rhPRG4 significantly delayed the scratch closure process in the absence or presence of TGFβ suggesting suppression of cell migration (**Fig 2C and 2D).** Collectively these results demonstrate that rhPRG4 potently inhibits the invasive and migratory properties of breast cancer cells *in vitro*.

### rhPRG4 does not appear to regulate TGFβ-Smad signalling

**Fig 3:**
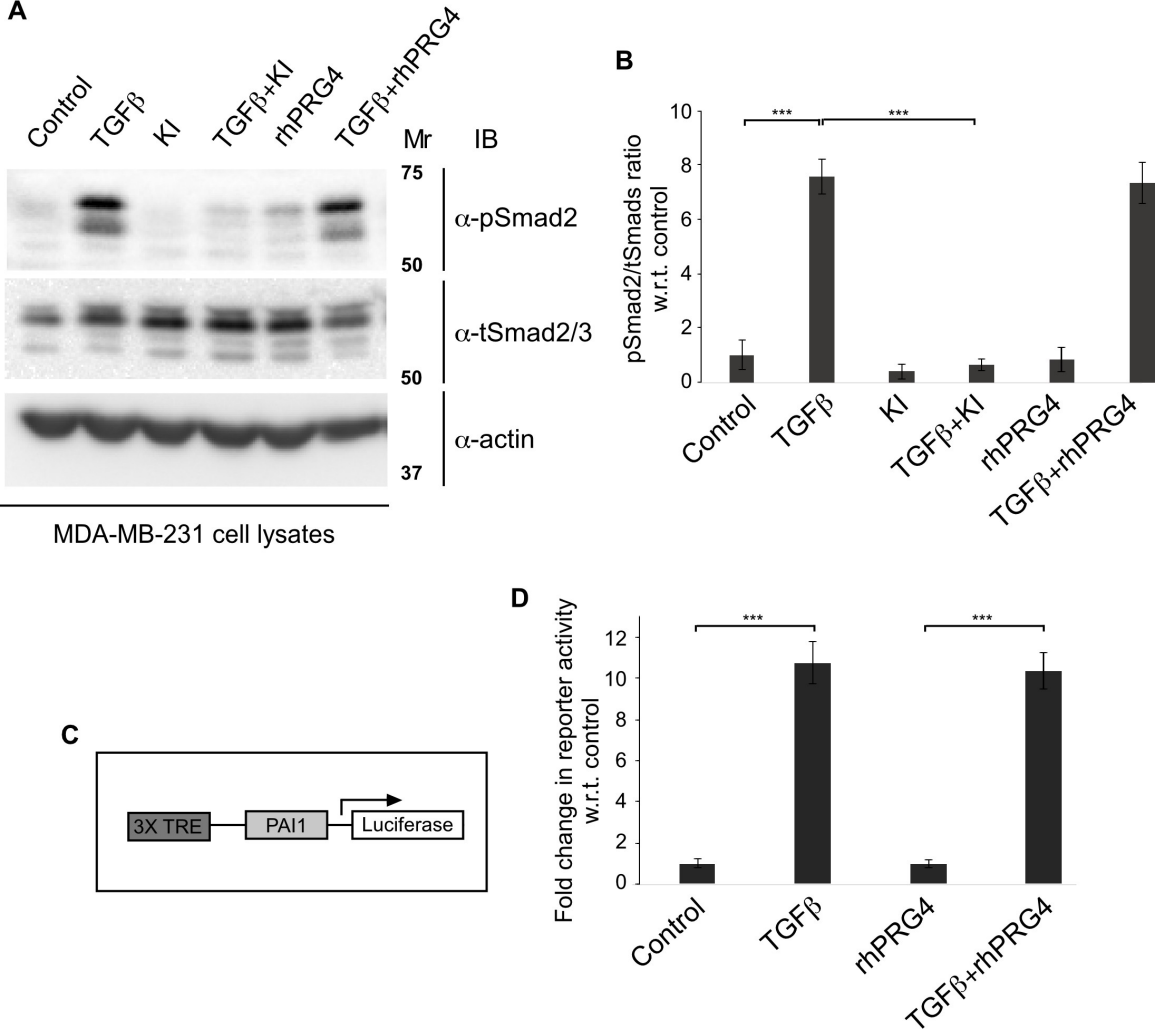
rhPRG4 does not affect TGFβ-Smad signalling. **(A)** phospho-Smad2 (pSmad2), total Smad2/3 (tSmad2/3) and actin immunoblots of lysates of MDA-MB-231 cells which were either left untreated (control) or incubated with 100 pM TGFβ, without or with 10 μM KI or 100 μg/mL rhPRG4 in complete growth medium for 12h. Mr indicates Markers’ molecular size. **(B)** Bar graph represents the mean ± SEM of the proportion of pSmad2 relative to the total protein abundance of total Smad2/3 and expressed as fold change with respect to the control from four independent experiments including the one shown in A. **(C)** A schematic representation of the 3TP-Lux reporter construct with three consecutive TPA (12-O-tetradecanoylphorbol 13-acetate) response elements (TREs) and a portion of the plasminogen activator inhibitor 1 (PAI-1) promoter region driving the expression of luciferase gene. TGFβ treatment triggers the phosphorylation, nuclear translocation, and binding of the Smads to 3TP promoter leading to increase in the abundance and hence activity of the luciferase enzyme. **(D**) MDA-MB-231 cells were transfected with the 3TP-Lux reporter construct along with a Renilla luciferase expression construct driven by a CMV promoter. Cells were left untreated (control) or incubated with 100 pM TGFβ, without or with 100 μg/mL rhPRG4 in 0.2% FBS-containing medium. Bar graph represents the mean ± SEM of the 3TP promoter-driven luciferase values normalized to the Renilla luciferase expression (relative light units), and the normalized data are expressed relative to the normalized luciferase data in lysates of untreated cells from three independent experiments. Significant difference, ANOVA: ***P ≤ 0.001.

Our findings that rhPRG4 promotes anti-invasive and anti-migratory effects raised the question how PRG4 achieves these cellular responses. As rhPRG4 supressed TGFβ-induced invasion and migration of MDA-MB-231 cells *in vitro*, we tested first the idea that rhPRG4 may antagonize TGFβ signalling pathway. Binding of TGFβ ligands to the cognate receptors on the cell surface, leads to the phosphorylation of receptor-regulated proteins Smad 2 and 3, which are important for downstream TGFβ signalling [32]. Thus, we tested if rhPRG4 affects the ability of TGFβ to induce the phosphorylation of Smad2 on its last C-terminal serine residues in these cells. Immunoblotting analyses of lysates of MDA-MB-231 cells, incubated with growth medium without or with TGFβ, alone or together with KI or rhPRG4, interestingly revealed that rhPRG4 did not appreciably alter TGFβ-induced phosphorylation of Smad2 on Serine residues 465 and 467 of the C-terminus (**Fig 3A and 3B)**.

Next, we carried out further analyses to ascertain if rhPRG4 affects TGFβ-Smad-dependent signalling. In particular, we determined the effect of rhPRG4 on TGFβ-Smad-induced transcriptional activity using the widely used 3TP-Lux reporter assay in which the expression of the firefly luciferase gene is under the control of three-tandem repeats of a TPA-responsive elements (3T) and promoter elements of the TGFβ-responsive gene the *plasminogen activator inhibitor 1* (PAI-1) [36]. MDA-MB-231 cells transfected with 3TP-lux plasmid together with a CMV-Renilla luciferase plasmid as an internal transfection efficiency control, were incubated overnight with low serum-containing growth medium without or with TGFβ, alone or with rhPRG4, lysed and then subjected to luciferase assays. As expected, TGFβ led to significant induction of 3TP-Lux reporter activity **(Fig 3C and 3D)**. However, rhPRG4 did not alter TGFβ-induced 3TP-Lux reporter activity in MDA-MB-231 cells. These data are consistent with the lack of effect of rhPRG4 on TGFβ-induced Smad2 phosphorylation. Collectively, these data suggest that rhPRG4 may act downstream of TGFβ-Smad-induced transcription to affect the biological processes of migration and invasion.

### rhPRG4 suppresses hyaluronan-induced invasion of breast cancer cells

**Fig 4:**
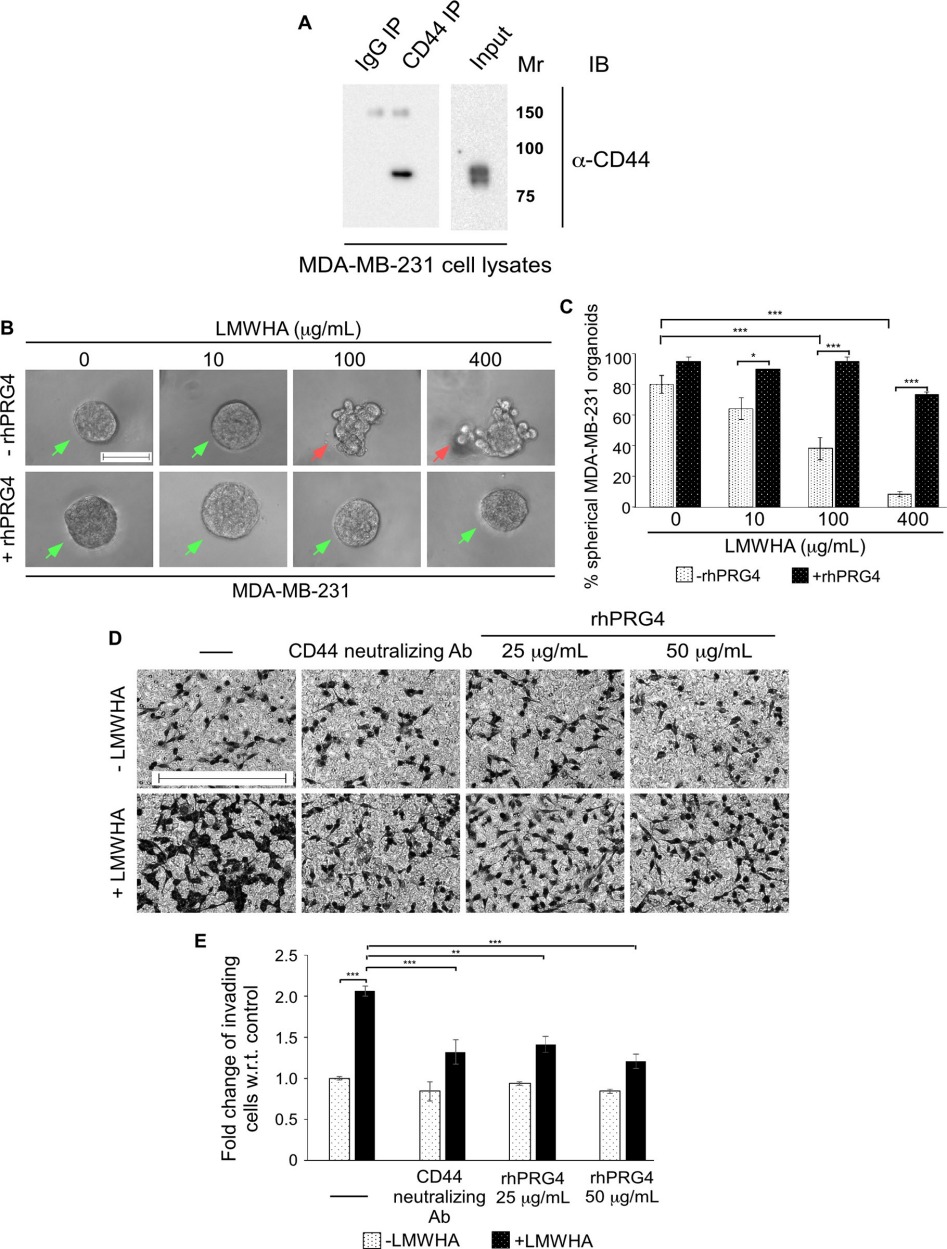
rhPRG4 suppresses low molecular weight hyaluronic acid (LMWHA)-induced invasion of breast cancer cells. **(A)** Lysates of MDA-MB-231 cells were subjected to immunoprecipitation using a rat CD44 antibody (CD44 IP) or a non-specific rat IgG antibody (IgG IP) followed by CD44 immunoblotting of the immunoprecipitates. CD44 protein abundance in the lysates was also confirmed by CD44 immunoblotting (input). Mr indicates Markers’ molecular size. **(B)** Representative DIC light microscopy images of 8-day old three-dimensional MDA-MB-231 cell-derived organoids incubated with growth medium without or with increasing concentrations of LMWHA (10, 100 or 400 μg/mL), alone or together with 100 μg/mL rhPRG4. Scale bar indicates 50 μm. Green arrows and red arrows indicate spherical and invasive organoids, respectively. **(C)** Bar graph depicts mean ± SEM proportion of spherical organoids expressed as a percentage of total colonies counted for each experimental condition from three independent experiments including the one shown in B. **(D)** Representative DIC light microscopy images of crystal violet-stained 12h-serum-starved MDA-MB-231 cells appearing on the underside of Matrigel-coated membrane of a transwell insert, with the bottom well containing complete growth medium without (-) or with 400 μg/mL LMWHA, alone or with 5 μg/mL CD44 neutralizing antibody, 25 μg/mL rhPRG4 or 50 μg/mL rhPRG4. Scale bar represents 150μm. **(E)** Bar graph depicts mean ± SEM fold change of invaded cells relative to control were counted from eight randomly chosen non-overlapping fields for each experimental condition from four independent experiments including the one shown in D. ANOVA: Significant difference, ANOVA: *P ≤ 0.05, **P ≤ 0.01, ***P ≤ 0.001.

Increasing evidence suggests that a key downstream signalling axis that contributes to TGFβ-induced invasion and metastasis of breast cancer is the hyaluronan (HA)-cluster of differentiation 44 (CD44) pathway [37–39]. A number of reports suggest that TGFβ can increase the abundance of hyaluronan synthase 2 (HAS2) enzyme that catalyzes the production and secretion of hyaluronan (HA), especially the low molecular weight hyaluronan (LMWHA) in the stroma [40,41]. In addition, TGFβ is suggested to increase the expression of the HA receptor CD44 in tumor cells [38]. In turn, evidence suggest that LMWHA associates with CD44, triggering the activation of specific signalling pathways that enhance invasion and metastatic ability of tumor cells [37–39]. Recent *in vitro* data support the idea that in addition to being a boundary lubricant and possessing anti-adhesive properties, rhPRG4 may compete with HA for CD44 binding, which may suppress downstream signalling, contributing to the proliferation of osteoarthritis- and rheumatoid arthritis-derived synoviocytes [9,10]. Given this evidence, we asked if rhPRG4 suppression of TGFβ-mediated invasive growth of 3D-breast cancer cell-derived organoid as well as invasiveness and migration of cells detected by transwell invasion and scratch healing assays **(Figs 1A, 2A and 2C)** involves disruption of a TGFβ-controlled HA-CD44 signalling axis. To address this question, we first characterized the steady-state protein level of CD44 in the MDA-MB-231 cells. Immunoprecipitation followed by immunoblotting analysis of cell lysates indicated that CD44 is expressed in the MDA-MB-231 cells **(Fig 4A)**, raising the possibility of an active CD44-dependent signalling axis. To address this question, we evaluated the effect of HA on the invasive behavior of the 3D-MDA-MB-231 cells. We used LMWHA (<10 kDa) which has been reported to be elevated in the breast tumor stroma and serum in metastatic disease [42,43]. The 3D-MDA-MB-231 cells were left untreated or incubated with increasing concentrations of LMWHA either alone or in combination with rhPRG4. LMWHA acted in a dose-dependent manner to increase the proportion of invasive organoids, and as reflected by the decrease in the proportion of spherical organoids (**Fig 4B and 4C**). However, rhPRG4 suppressed the ability of LMWHA to promote the invasive growth of breast cancer cell-derived organoids. Consistent with the results from the 3D-cultures, in transwell invasion assays, we found that LMWHA increased the proportion of invading cells (**Fig 4D** and **4E**). On the other hand, a CD44 neutralizing antibody which interferes with the HA-CD44 interaction [24], also blocked cell invasion in the presence of LMWHA. Similarly, rhPRG4 was able to inhibit breast cancer cell invasion in the presence of exogenous LMWHA. Collectively, these results indicate that LMWHA induces invasive behaviour in MDA-MB-231 cells, both in 3D culture and transwell invasion assay, which can be blocked by rhPRG4.

### CD44 is crucial for TGFβ-induced invasiveness in MDA-MB-231 cells

**Fig 5:**
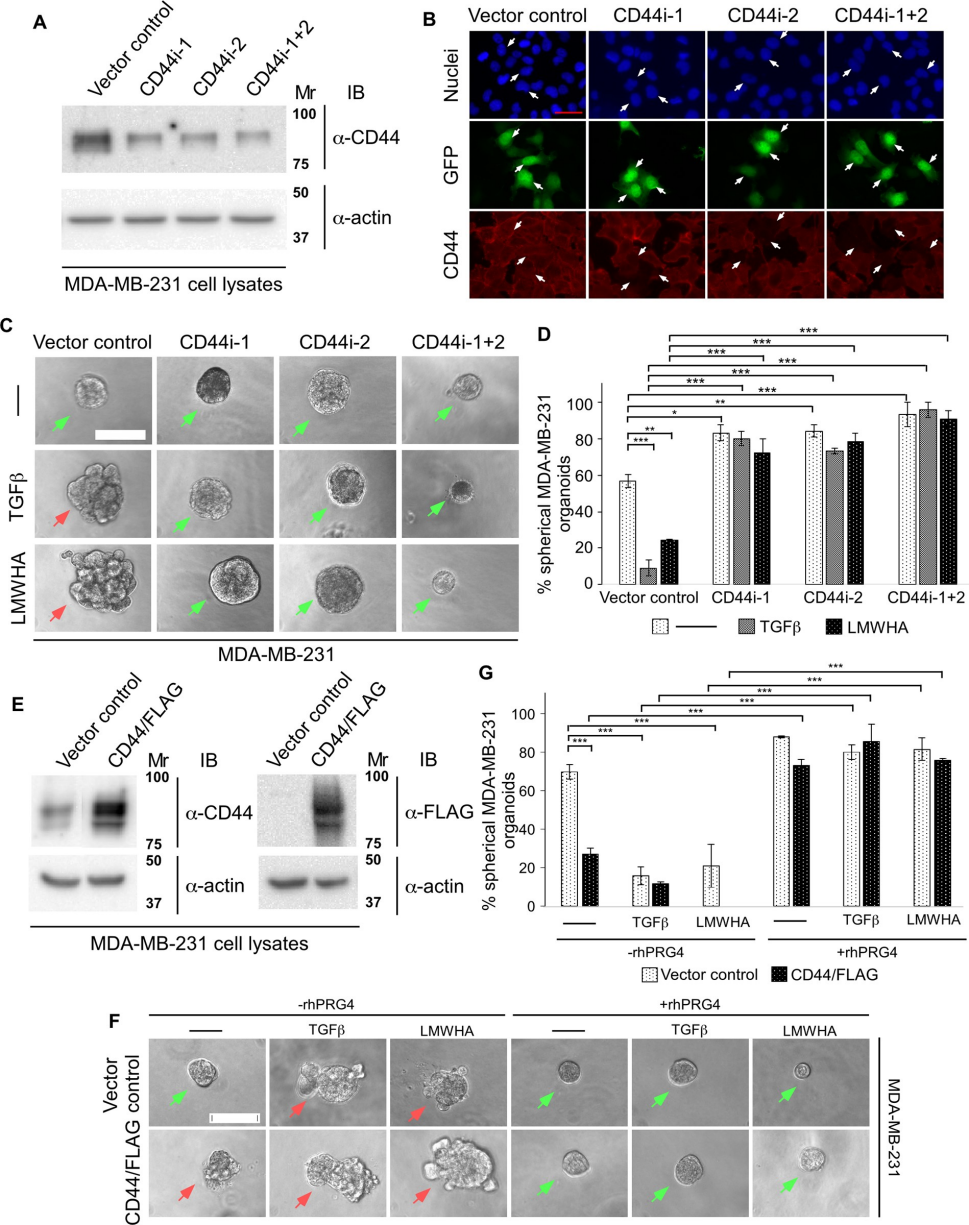
CD44 is crucial for TGFβ-induced invasiveness in MDA-MB-231 cells. **(A)** CD44 immunoblot of lysates of MDA-MB-231 cells transfected with the pU6 RNAi vector (vector control), or the plasmids CD44i-1, CD44i-2, alone or together (CD44i-1+2) that expresses shRNAs targeting two distinct sequences of CD44 mRNA. Actin was used as loading control. **(B)** Representative CD44, GFP and nuclei fluorescence microscopy images of MDA-MB-231 cells transfected as in A, and subjected to anti-CD44 indirect immunofluorescence (rat anti-CD44/anti-rat Alexa 647, red) and counterstained with Hoechst 33342 fluorescent nucleotide dye (blue) to visualize nuclei. GFP (green) signal indicate vector control or CD44 RNAi-1/2-transfected cells. Arrows show examples of vector (left), CD44i-1 (2^nd^ column), CD44i-2 (3^rd^ column) and CD44i-1+2 (right) transfected cells to highlight the knockdown of endogenous CD44 by the two CD44 RNAi plasmids. This experiment was repeated three times with similar outcomes. **(C)** Representative DIC light microscopy images of untreated (-), 100 pM TGFβ or 400 μg/mL LMWHA-treated 8-day old three-dimensional organoids in complete growth medium, derived from MDA-MB-231 cells, transfected with vector control or CD44i-1 and CD44i-2, individually or in combination. **(D)** Bar graph depicts mean ± SEM proportion of spherical organoids expressed as a percentage of total colonies counted for each experimental condition from three independent experiments including the one shown in C. **(E)** CD44 and FLAG immunoblots of lysates of MDA-MB-231 cells transfected with an empty vector or CD44/FLAG expression plasmids. Actin was used as loading control. **(F)** Representative DIC light microscopy images of vector control or CD44/FLAG expressing 8-day old MDA-MB-231 cell-derived organoids grown in complete growth medium without (-) or with 100 pM TGFβ or 400 μg/mL LMWHA, alone or with 100 μg/mL rhPRG4. **(G)** Bar graph depicts mean ± SEM proportion of spherical organoids expressed as a percentage of total colonies counted for each experimental condition from three independent experiments including the one shown in F. Significant difference, ANOVA: *P ≤ 0.05, **P ≤ 0.01, ***P ≤ 0.001. Mr indicates Markers’ molecular size. Scale bar indicates 50 μm. Green arrows and red arrows indicate spherical and invasive organoids, respectively.

To further investigate whether TGFβ- and LMWHA-mediated invasiveness of the MDA-MB-231 cells are CD44-dependent, we next used an RNA interference (RNAi) approach of gene silencing to knockdown CD44 in these cells. Two small hairpin RNAs (shRNAs) were designed targeting specific sequences in exon 16 and exon 4 of CD44, respectively. CD44 immunoblotting of lysates of MDA-MB-231 cells transfected with a control pU6 RNAi vector or RNAi plasmid expressing shRNA1, shRNA2, alone or together confirmed that these shRNAs individually or together efficiently knocked down endogenous CD44 (**Fig 5A)**. The reduction in the protein abundance of endogenous CD44 by shRNA1, shRNA2, alone or together, was significant as compared to control (**S2A Fig**). CD44 immunofluorescence analysis of fixed MDA-MB-231 cells transfected with the RNAi control vector or a plasmid expressing CD44i-1/2 showed that CD44i-1/2 produced efficient reduction in the protein abundance of endogenous CD44 **(Fig 5B).**

Next, we tested the effect of endogenous CD44 knockdown by CD44 shRNA1/2 on 3D-MDA-MB-231 cell-derived organoids in the presence or absence of TGFβ or LMWHA. Knockdown of CD44 increased the percentage of spherical organoids, and suppressed the ability of TGFβ or LMWHA to induce an invasive behavior as demonstrated by preservation of spherical organoids (**Fig 5C and 5D**). Interestingly, coexpression of CD44i-1 and CD44i-2, reduced the size of the organoids in the absence or presence of TGFβ or HA (**Fig 5C** and **S2B Fig**). These data suggest that CD44 may mediate TGFβ and LMWHA-induction of invasive growth of MDA-MB-231 cell-derived organoids.

Having established the importance of the receptor CD44 for TGFβ and LMWHA-induced invasiveness in MDA-MB-231-derived organoids, we next determined the effect of overexpression of CD44 on these organoids in the absence or presence of TGFβ and HA. First, we RT-PCR-amplified an open reading frame of CD44 cDNA from the MDA-MB-231 cells and subcloned into a CMV-based plasmid to express CD44/FLAG in MDA-MB-231 cells which was confirmed by CD44 and FLAG immunoblotting (**Fig 5E)**. 3D-organoids were generated from MDA-MB-231 cells transiently transfected with a vector control or with a CD44/FLAG expressing plasmid, and were incubated with growth medium without or with TGFβ, LMWHA, alone or together with rhPRG4. Untreated 3D-organoids derived from vector control transfected cells were mostly spherical and became invasive upon incubation with TGFβ or LMWHA whereas these effects were significantly reversed by rhPRG4 **(Fig 5F and 5G**). However, overexpressed CD44/FLAG promoted invasive growth of the 3D-organoids even in the absence of TGFβ or LMWHA. rhPRG4 suppressed the ability of overexpressed CD44 to promote invasive growth of MDA-MB-231 cell-derived organoids in the absence or presence of TGFβ or LMWHA. These results further support the notion that rhPRG4 suppresses breast cancer cell invasive growth in a CD44-dependent manner. Altogether, findings from CD44 knockdown and overexpression studies suggest that rhPRG4 suppresses CD44-mediated TGFβ or LMWHA promotion of an invasive phenotype of the MDA-MB-231 cell-derived organoids.

### rhPRG4 supresses TGFβ- and LMWHA-mediated invasiveness of HCC38 cell-derived organoids in a CD44-dependent pathway

**Fig 6:**
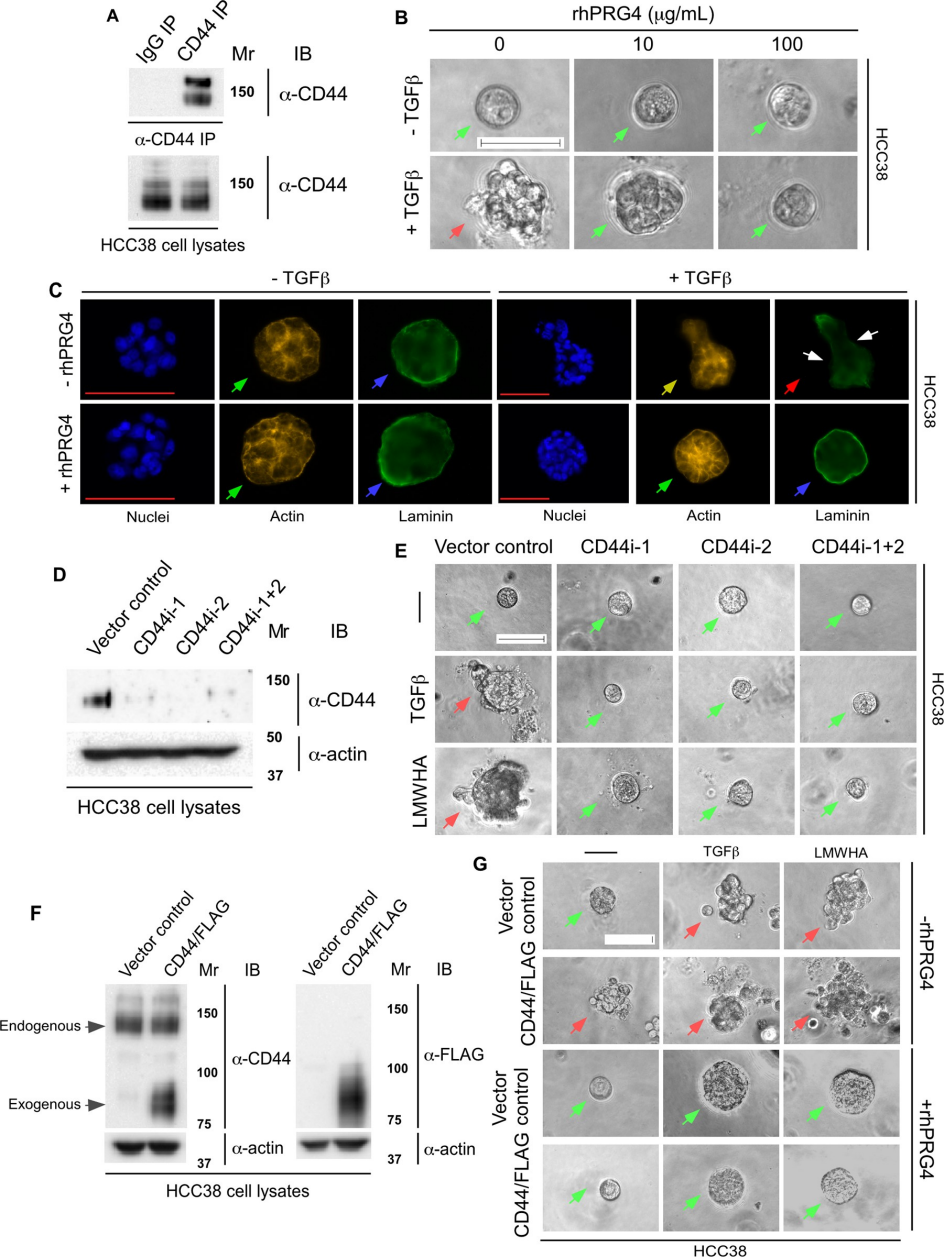
rhPRG4 acts in a CD44-dependent manner to supress TGFβ- or LMWHA-induced invasive growth of HCC38. **(A)** Lysates of HCC38 cells were subjected to immunoprecipitation using a mouse CD44 antibody (CD44 IP) or a non-specific mouse IgG antibody (IgG IP) followed by CD44 immunoblotting. The protein abundance of CD44 in the lysates was confirmed by CD44 immunoblotting. **(B)** Representative DIC light microscopy images of 6-day old three-dimensional HCC38 cell-derived organoids that were left untreated or incubated with 100 pM TGFβ, without or with increasing concentrations of rhPRG4 (10 and 100 μg/mL) in complete growth medium. **(C)** Representative fluorescence microscopy images of nuclear (Hoechst 33342, blue), actin (TRITC- phalloidin, yellow), and laminin (rat anti-laminin/anti-rat Alexa 488, green) staining of formaldehyde-fixed 6-day old HCC38 cell-derived organoids that were left untreated or incubated with TGFβ, with or without rhPRG4, in complete growth medium. This experiment was repeated two independent times with similar outcomes. **(D)** CD44 immunoblotting of lysates of HCC38 cells transfected with the pU6 RNAi vector (vector control), or the plasmids CD44i-1, CD44i-2, alone or together (CD44i-1+2) expressing shRNAs targeting two distinct sequences of CD44 mRNA. Actin was used as loading control. **(E)** Representative DIC light microscopy images of untreated (-), 100 pM TGFβ or 400 μg/mL LMWHA-treated 6-day old three-dimensional organoids derived from HCC38 cells transfected with vector control or CD44i-1, CD44i-2, individually or in combination. **(F)** CD44 and FLAG immunoblotting of lysates of HCC38 cells transfected with an empty vector or CD44/FLAG expression plasmid. Actin was used as loading control. **(G)** Representative DIC light microscopy images of vector control or CD44/FLAG expressing 6-day old HCC38 cell-derived organoids grown in complete growth medium without (-) or with 100 pM TGFβ or 400 μg/mL LMWHA, alone or with 100 μg/mL rhPRG4. Mr indicates Markers’ molecular size. Scale bar indicates 50μm. For Fig 6B, 6E and 6G green arrows and red arrows indicate spherical and invasive organoids, respectively. For Fig 6C, green arrows indicate cortical actin, yellow arrow indicates stress-fibre like actin, blue arrows indicate intact laminin rings, red arrow indicates disruption of laminin ring, and white arrows indicate representative localized sites of laminin loss.

To further confirm that rhPRG4 acts via CD44 to inhibit invasive behaviour of TNBC cells, we investigated this phenomenon in another CD44-expressing TNBC cell, namely HCC38 cells. Immunoprecipitation followed by immunoblot analysis of HCC38 cell lysates revealed a 150kDa CD44-immunoreactive specific band as the major CD44 protein species (**Fig 6A**). Culturing of HCC38 cells in the context of Matrigel led to the formation of small smooth-surfaced spherical multicellular structures (**Fig 6B** and **S3A Fig**). TGFβ led to deformation of the organoids characterized by invasive behaviour. rhPRG4 acted in a dose-dependent manner to promote a spherical-smooth phenotype even in the presence of TGFβ. Immunofluorescence analyses of these organoids suggested, similar to MDA-MB-231, the presence of cortical actin organization and intact laminin rings surrounding the untreated control organoids cultures. TGFβ led to stress-fibre like reorganization, and laminin ring loss at the invasive front of the organoids suggesting localized basement membrane breach (**Fig 6C)**. Importantly, rhPRG4 suppressed these effects of TGFβ, thus maintaining sphericity, cortical actin organization, and intact laminin ring surrounding the organoids.

Loss and gain of function of CD44 in these cells, acted similar to MDA-MB-231 cells. Reduction of CD44 protein by shRNA1, shRNA2, alone or together, maintained the spherical nature of organoids even in presence of TGFβ or LMWHA, suggesting the importance of CD44 for TGFβ and LMWHA-promoted invasion of 3D-HCC38 cell-derived organoids (**Fig 6D and 6E** and **S3B and S3C Fig**). Conversely, overexpression of CD44 in these cells promoted an invasive phenotype of the organoids even in the absence of TGFβ or LMWHA (**Fig 6F and 6G** and **S3D Fig**). rhPRG4 suppressed the ability of overexpressed CD44 to promote an invasive phenotype of the 3D-HCC38 cell-derived organoids in the absence or presence of TGFβ or LMWHA, thus maintaining a spherical shape. Altogether, these data suggest that rhPRG4 opposes the ability of TGFβ and LMWHA to induce invasive growth of TNBC cells in a CD44-dependent manner.

### HA-CD44 axis mediates TGFβ-induced invasive growth of breast cancer cell-derived organoids

**Fig 7:**
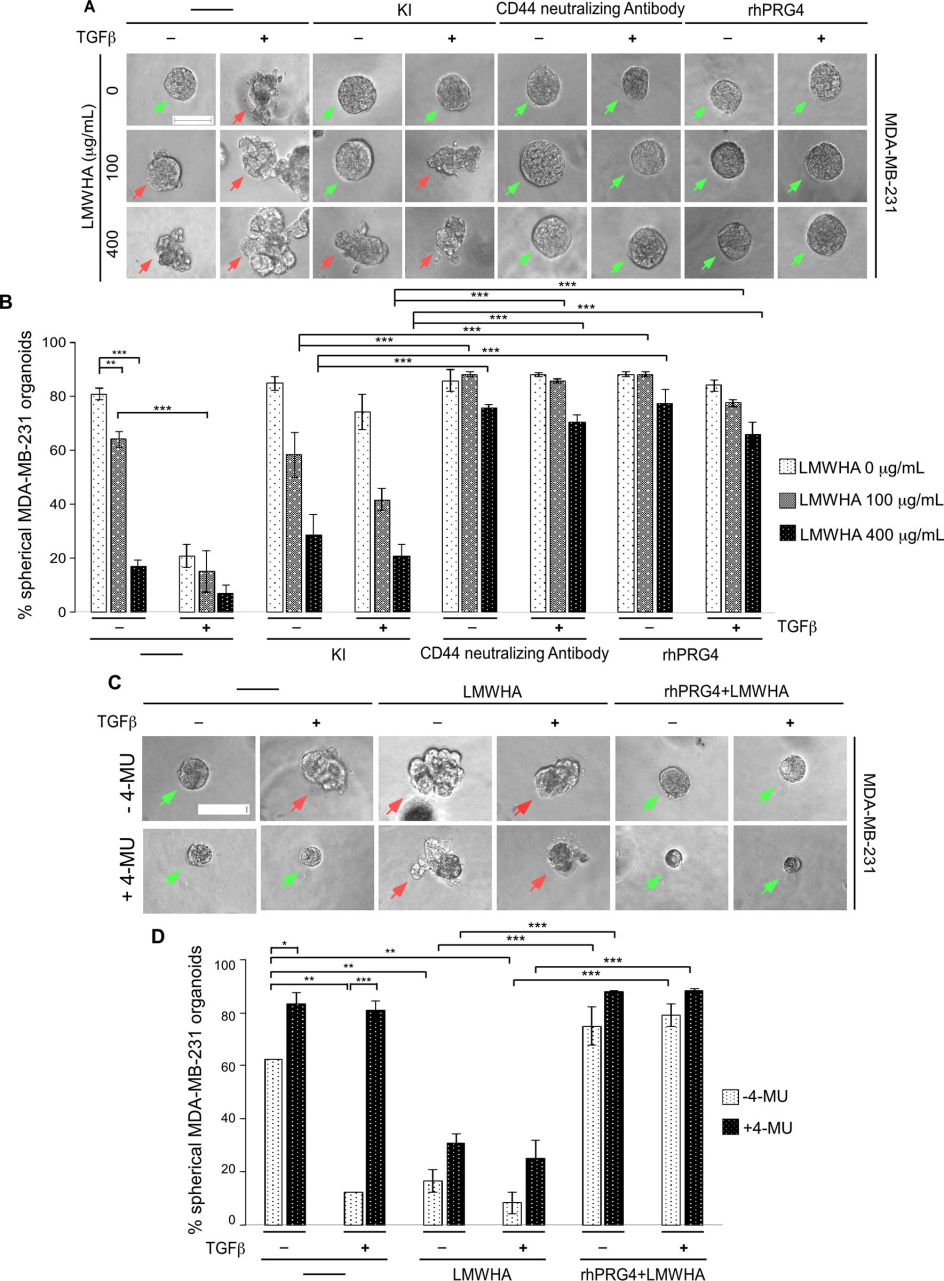
TGFβ induces invasiveness in the breast cancer cells in a HA-CD44-dependent manner. **(A)** Representative DIC light microscopy images of 8-day old three-dimensional MDA-MB-231 cell-derived organoids that were left untreated (-) or incubated with different concentrations of LMWHA (100 or 400 μg/mL), without or with 100 pM TGFβ, along with or without KI (10 μM), CD44 neutralizing antibody (2.5 μg/mL), or rhPRG4 (100 μg/mL), in complete growth medium. **(B)** Bar graph depicts mean ± SEM proportion of spherical organoids expressed as a percentage of total organoids counted for each experimental condition from three independent experiments including the one shown in A. **(C)** Representative DIC light microscopy images of 8-day old three-dimensional MDA-MB-231 cell-derived organoids that were treated without or with 0.5 mM 4-MU, without or with 100 pM TGFβ, along with or without 400 μg/mL LMWHA, alone or together with 100 μg/mL rhPRG4 in complete growth medium. **(D)** Bar graph depicts mean ± SEM proportion of spherical organoids expressed as a percentage of total colonies counted for each experimental condition from three independent experiments including the one shown in C. Significant difference, ANOVA: *P ≤ 0.05, **P ≤ 0.01, ***P ≤ 0.001. Scale bar indicates 50 μm. Green arrows and red arrows indicate spherical and invasive organoids, respectively.

To further investigate the role of CD44 in TGFβ-induced invasive growth of MDA-MB-231 cell-derived organoids, 3D cultures were incubated with growth medium or increasing concentrations of LMWHA, alone or together with different combination of TGFβ, KI, CD44 neutralizing antibody or rhPRG4 (**Fig 7A and 7B**). Consistent with the previous findings, incubation of 3D-MDA-MB-231 cell-derived cultures with increasing concentrations of LMWHA promoted an invasive growth of the breast cancer cell-derived organoids. TGFβ further enhanced the ability of LMWHA to induce invasive growth of these organoids. Interestingly, KI which suppressed the TGFβ-induced invasive growth, could not reverse the LMWHA effect, suggesting LMWHA-mediated invasion acts downstream of the TGFβ signalling pathway. However, the CD44 neutralizing antibody suppressed the ability of both LMWHA and TGFβ stimuli to induce invasive growth, and indeed this intervention significantly increased the number of spherical organoids compared to KI treatment. Furthermore, the blockade of TGFβ-induced invasive growth of these organoids by CD44 neutralizing antibody suggests that TGFβ induces invasiveness in a CD44-dependent pathway in these cells. PRG4, similar to the CD44 antibody, suppressed TGFβ and LMWHA-induced invasive growth of the MDA-MB-231 cell-derived organoids. These data suggest that rhPRG4 is working downstream of both the LMWHA and TGFβ-mediated pathways. Collectively, these results indicate that the HA-CD44 axis contributes significantly to TGFβ-induced invasive growth of MDA-MB-231 cells and this signalling axis appears to be a rhPRG4 target.

To further determine the impact of HA-CD44 pathway on TGFβ-induced invasiveness of these cells, and more specifically the role of HA synthesis by the hyaluronic acid synthase (HAS) enzymes [44], we examined the effect of 4-methylumbelliferone (4-MU, a HAS inhibitor) on 3D-MDA-MB-231 cell-derived organoids [45]. Specifically, 3D-MDA-MB-231 cell-derived organoids were incubated without or with 4-MU, alone or together with TGFβ, with different combinations of LMWHA and rhPRG4 (**Fig 7C and 7D**). 4-MU supressed TGFβ-induced invasive growth of 3D-MDA-MB-231 cell-derived organoids, which was reversed by addition of exogenous LMWHA. Conversely, rhPRG4 suppressed LMWHA-induced invasive growth of 4-MU-treated 3D-MDA-MB-231 cell-derived organoids. Collectively, these data indicate that TGFβ induces invasive growth of 3D-MDA-MB-231 cell-derived organoids in an HA-dependent manner. Furthermore, rhPRG4 suppression of both TGFβ and LMWHA-induced invasive growth of 3D-MDA-MB-231 cell-derived spheroids suggest that rhPRG4 acts downstream of HA production and its signalling pathways.

Overall, these results suggest an interplay between TGFβ signalling and HA-CD44 axis in promoting the invasive behaviour of 3D-MDA-MB-231 cell-derived organoids. Importantly, our study suggests that rhPRG4 may act downstream of TGFβ to suppress HA-CD44 axis's ability to promote invasive growth of the MDA-MB-231-derived organoids.

### HA-CD44 signalling axis is regulated by TGFβ and PRG4

**Fig 8:**
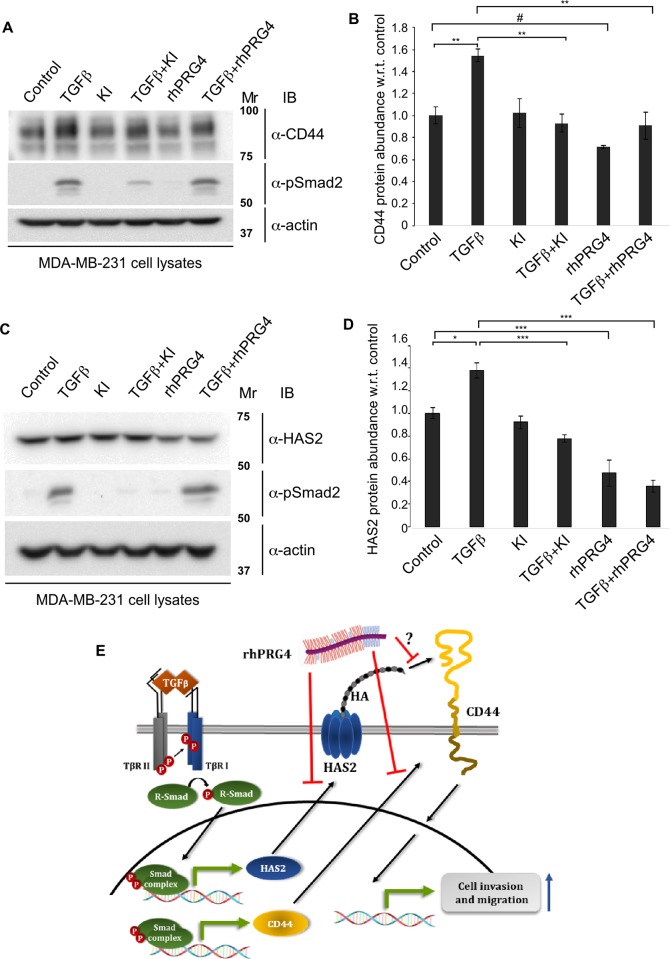
rhPRG4 and TGFβ have opposing effects on the protein abundance of CD44 and HAS2. **(A)** CD44 and phospho-Smad2 (pSmad2) immunoblots of lysates of MDA-MB-231 cells incubated in complete growth medium without (control) or with 100 pM TGFβ, alone or together with 10 μM KI or 100 μg/mL rhPRG4. Actin was used as loading control. **(B)** Bar graph depicts mean ± SEM proportion of CD44 immunoreactive band in each treatment condition from four independent experiments including the one shown in A. **(C)** HAS2 and phospho-Smad2 (pSmad2) immunoblots of lysates of MDA-MB-231 cells incubated in complete growth medium without (control) or with 100 pM TGFβ, alone or together with 10 μM KI or 100 μg/mL rhPRG4. Actin was used as loading control. **(D)** Bar graph depicts mean ± SEM proportion of HAS2 immunoreactive band in each treatment condition from four independent experiments including the one shown in D. **(E)** A schematic diagram showing the relationship amongst TGFβ, HA-CD44 signalling and rhPRG4 in MDA-MB-231 cells, uncovered in this study. TGFβ increases the protein abundance of HAS2 and CD44 to enhance cancer cell’s invasion and migration. rhPRG4 does not alter Smad phosphorylation but decreases HAS2 and CD44 protein abundance leading to suppression of invasion and migration of cancer cells. rhPRG4 may interfere with HA-CD44 interaction. Mr indicates Markers’ molecular size. Significant difference, ANOVA: *P≤ 0.05, **P≤ 0.01, ***P ≤ 0.001;; unpaired T test: ^**#**^P = 0.0164.

To gain further insight into the molecular mechanisms by which TGFβ may regulate CD44 signalling, we determined the effect of TGFβ on the protein abundance of CD44 in MDA-MB-231 cells using immunoblotting analyses. These experiments showed that activation of TGFβ-signalling promoted the protein abundance of CD44 in MDA-MB-231 cells (**Fig 8A and 8B)**. In contrast, we found that PRG4 reduced the protein abundance of CD44 in the absence or presence of TGFβ. rhPRG4 did not reduce TGFβ-induced Smad2 phosphorylation suggesting that rhPRG4-mediated CD44 suppression may not involve this step of the pathway. In other analyses using CD44 indirect immunofluorescence of untreated, TGFβ and/or PRG4-treated MDA-MB-231 cell-derived organoids revealed that TGFβ enhanced the CD44-immunostaining signal, whereas rhPRG4 reduced this signal in the absence or presence of TGFβ, thus further confirming the immunoblotting data (**S4 Fig)**.

That the HAS2 inhibitor 4-MU suppressed TGFβ-induced invasiveness of 3D-breast cancer cell-derived organoids suggested that TGFβ may regulate the production of HA in the MDA-MB-231 cells. Thus, we examined the effect of TGFβ on HAS protein levels. Amongst HAS1/2/3 isoforms, HAS2 is the most prevalent enzyme in MDA-MB-231 cells [46]. Immunoblotting analyses showed that TGFβ increased the protein abundance of HAS2 in MDA-MB-231 cells (**Fig 8C and 8D)**. Interestingly, rhPRG4 suppressed the protein abundance of HAS2 in the absence or presence of TGFβ.

These analyses reveal that in contrast to TGFβ, rhPRG4 leads to reduction in the protein abundance of CD44 and HAS2 in the absence or presence of exogenous TGFβ, which could provide a mechanism by which rhPRG4 suppresses TGFβ-induced invasiveness of breast cancer cells.

## Discussion

In this study, we have uncovered novel anti-migratory and anti-invasive roles for the mucin-like glycoprotein rhPRG4 in carcinoma cells derived from patients with triple-negative breast cancer (TNBC). In particular, by counteracting the ability of the cytokine TGFβ to promote invasive growth of the three-dimensional human MDA-MB-231 and HCC38 TNBC cell-derived organoids, rhPRG4 preserves a non-invasive spherical morphology of these multicellular structures. Epistatic studies revealed that rhPRG4 acts downstream of TGFβ-Smad signalling to achieve its anti-migratory and anti-invasive effects. Moreover, our data suggest that rhPRG4 disrupts TGFβ-induced HA-CD44 signalling activation, which plays a key role in the invasive growth of MDA-MB-231 cell-derived organoids by this cytokine. In biochemical analyses, we find that TGFβ signalling increases, while rhPRG4 reduces, in the absence or presence of exogenous TGFβ, the protein abundance of the HA-producing enzyme HAS2 and the HA receptor CD44 in MDA-MB-231 cells, thus pointing to a mechanism by which rhPRG4 inhibits invasion and migration in these cells. Altogether, our findings add important insights into the biological functions and potential therapeutic implications of rhPRG4.

While PRG4 is more of a mucin-like glycoprotein versus a classic proteoglycan per se, other proteoglycans can act directly or indirectly to affect multiple signalling axes, including the TGFβ pathway, leading to positive or negative regulation of multiple cellular processes such as angiogenesis, cell migration and invasion of diverse types of cancer cells and in a context-dependent manner [47,48]. However, to our knowledge, our findings that rhPRG4 exerts anti-migratory and anti-invasive effects in breast cancer cells is the first report to demonstrate a role for this glycoprotein in epithelial tissue-derived cancers, which represent the majority of solid tumors [2]. The relevance of these newly identified rhPRG4’s biological roles in controlling metastasis, which is the major obstacle in cancer treatment, should pave the way for future studies to explore if rhPRG4 can display anti-metastatic potential. In addition to the cancer relevance, our findings contribute important evidence to the growing idea that rhPRG4 can regulate cellular responses thus acting beyond its original identified role as purely a cartilage boundary lubricant. For example, data suggest that PRG4 has anti-inflammatory effects (e.g. decrease in expression of a number of pro-inflammatory cytokines and matrix remodelling enzymes, in a CD44 and toll-like receptor 2- and 4-dependent manner) with implications for osteoarthritis, rheumatoid arthritis and gout [9–13]. Our finding regarding the role of rhPRG4 in breast cancer adds a new dimension to the emerging biotherapeutic activity of the recombinant glycoprotein.

The finding that anti-PRG4 mAb 4D6, which specifically recognizes PRG4 based on immunoblotting and immunohistochemistry analyses [25,26], interferes with the ability of rhPRG4 to suppress invasive growth of breast cancer cell-derived organoids suggests that mAb 4D6 acts as an PRG4-neutralizing antibody. This previously unreported anti-PRG4 activity might reflect a hindrance by mAb 4D6 of PRG4 interactions with other molecules with relevance to its anti-invasive activity. Future identification of the epitope in PRG4 targeted by this antibody may add insight into functional domains of rhPRG4 with respect to its anti-invasive properties.

TGFβ plays a dual role in cancer initiation and progression [32,49]. At initial stages of neoplastic disease, evidence suggest that TGFβ acts as a tumor suppressor, while at the later stages of cancer, it can promote invasiveness and metastasis of different carcinomas including breast [32,49]. Thus, identifying ways to downregulate the tumor promoting role of TGFβ without affecting its tumor suppressive property may further control tumor growth. The finding that rhPRG4 suppresses TGFβ-induced invasive growth without affecting phosphorylation and the transcriptional activity of the receptor-regulated Smads (R-Smad, e.g. Smad2) raises the possibility that the ability of TGFβ to suppress tumor growth might be intact, which can be the subject of future investigations. That rhPRG4 anti-invasive actions on the MDA-MB-231 or HCC38-derived organoids are mediated by blockade of a LMWHA-CD44 signalling axis may have *in vivo* relevance. In general, enrichment of the cell surface glycoprotein CD44 in tumor cells including breast is correlated with invasive and metastatic characteristics of the cancer and hence poor prognosis [50]. HA, which is elevated in different carcinomas including breast cancer stroma [51] and blood serum [42,43] acts as a ligand for CD44. Depending on the number of the disaccharides repeats, HA is generally classified as high molecular weight hyaluronic acid (HMWHA) and LMWHA [40]. Importantly, LMWHA-CD44 binding can trigger activation of distinct signalling pathways that ultimately promote cancer cell invasion, migration and proliferation [38,52–54]. In addition, LMWHA-CD44 clusters can act to induce remodelling of the stromal ECM at the invasive front of a tumor mass [55]. The novel finding here that exogenous LMWHA promotes an invasive growth of MDA-MB-231 and HCC38 cell-derived organoids, is consistent with the idea thatelevated LMWHA in the tumor stroma can promote cancer invasiveness [51]. The idea that rhPRG4 negatively affects HA-CD44-induced invasiveness of cancer cells is consistent with other studies suggesting that PRG4 antagonizes HA-CD44-mediated inflammatory signalling that induce synoviocyte proliferation in rheumatoid arthritis and osteoarthritis diseases and a number of inflammatory cytokine production in human and murine macrophages [9,10,12]. Overall, these findings are consistent with the idea that rhPRG4 and LMWHA compete for CD44 binding [10] and extend them to breast cancer cells and the TGFβ pathway. Future studies could examine rhPRG4’s anti-invasive effects in other types of cancer, in particular high CD44-expressing cancer cells in HA-rich stromal environment [40,50].

Our study of rhPRG4 links TGFβ to HA-CD44 pathway activation in promoting breast cancer invasion, which is in agreement with previous findings [38]. It has been shown that TGFβ promotes the expression of HAS enzymes, particularly HAS2, which results in the accumulation of high levels of HA in the ECM of breast cancer cells [40,41]. Finally, TGFβ has also been shown to promote the expression of CD44 in a Smad-dependent manner [38,56]. In this study, we report that TGFβ increases the protein abundance of both HAS2 and CD44 in MDA-MB-231 cells. Collectively, these results, together with the the gain and loss of function analyses of HAS2 and CD44 loss of function analyses, demonstrate that the HA-CD44 pathway plays a key role in mediating the ability of TGFβ to induce invasion and migration of these cells.

rhPRG4’s suppression of the ability of overexpressed CD44 to induce invasive growth of the MDA-MB-231 cell-derived organoids may involve reduction of HA-CD44 interaction, and/or by reduction of the protein abundance of CD44 and HAS2 as indicated by immunoblotting and immunofluorescence analyses. Our results demonstrated that rhPRG4 can reduce the TGFβ-induced upregulation of CD44 and HAS2 protein abundance in MDA-MB-231 cells without affecting the phosphorylation of Smad2, suggesting that rhPRG4 is operating downstream of TGFβ-Smad signalling pathway (**Fig 8E**). Whether rhPRG4 supresses TGFβ-induced HA-CD44 signalling axis by supressing MAPK activation in these cells, as suggested by previous literature [38], remains to be elucidated. Moreover, whether rhPRG4 competes with HA for CD44 binding in breast cancer cells, as shown in a previous study with a different cell type [10], requires further investigation. Whatever the underlying mechanism by which rhPRG4 inhibits TGFβ-HA-CD44-induced invasion of TNBC breast cancer cell might be, it is evident that rhPRG4 can significantly supress both TGFβ and LMWHA-induced invasiveness of MDA-MB-231 cells.

## Conclusions

In summary, our data demonstrate that rhPRG4 can supress TGFβ-induced invasion and migration of TNBC cells *in vitro*, at least in part through suppression of the ability of HA-CD44 signalling axis to mediate TGFβ stimuli. Our findings also demonstrate that rhPRG4 can antagonize TGFβ-induced increase in the protein abundance of CD44 and HAS2, which may explain its suppression of TGFβ-induced invasiveness of these cells. Lastly, rhPRG4 also can inhibit LMWHA-induced invasiveness of these cells. Given that TNBC aggressiveness and mortality is correlated with high CD44 expression [57], and previous therapeutic strategies which target TGFβ and CD44 signalling axes have shown promise yet with adverse side effects [40,58], along with the potential for rapid translational update of rhPRG4 for clinical evaluation, rhPRG4 represents an ideal candidate as a potential biological anti-cancer therapy. In conclusion, these findings contribute to the understanding of PRG4’s biological activity in the previously uninvestigated area of breast cancer, and provide the framework for future investigation to target not only breast cancer but potentially other cancers which are dependent on TGFβ and HA-CD44 signalling for their survival, proliferation and metastatic ability [40,50].

## Supporting information

S1 Fig Low-serum condition downregulates increase in MDA-MB-231 cell numbers. 2.5x10^5 MDA-MB-231 cells in 10% FBS-contaning growth medium were seeded per well of a 12-well tissue culture plate (0h). At 24h, medium was replaced with fresh DMEM with 10% FBS (full serum) or 0.2% serum (serum starve/low serum), followed by introducing a scratch per well (in all the wells) 24h later (or 48h post seeding). Cells were incubuated with regular or low serum medium, with and without TGFβ, KI or rhPRG4 in different combinations and left for another 36h post-scrach (or 84h post cell seeding). For each experiment, cell counts were obtained at the time points depicted in the graph, and average cell number ± SEM at each condition or timepoint from 3 independent experiments is plotted on y-axis versus time point on the x-axis.
(TIF)

S2 Fig CD44 shRNAs reduce endogenous CD44 in MDA-MB-231 cells with implications for 3D-cell-derived organoid size. **(A)** Bar graph depicts mean ± SEM proportion of CD44 immunoblot-derived signals of lysates of MDA-MB-231 cells transfected with the pU6 RNAi vector (vector control), CD44 shRNA expressing plasmid CD44i-1 or CD44i-2, or in combination (CD44i-1+2), from experiments that were repeated three independent times including the one shown in Fig 5A. **(B)** Bar graph depicts mean ± SEM proportion of surface area of organoids derived from MDA-MB-231 cells transfected with the pU6 RNAi vector (vector control), or a combination of the CD44i-1and CD44i-2 plasmids (CD44i-1+2), before subjecting to 3D culturing, and leaving untreated (-) or incubating with 100 pM TGFβ or 400μg/mL of LMWHA, from experiments that were repeated three independent times including the one shown in Fig 5C.
(TIF)

S3 Fig rhPRG4 suppresses CD44-dependent TGFβ- and LMWHA- induced invasive growth of 3D-HCC38 cell-derived organoids. **(A)** Bar graph depicts mean ± SEM proportion of spherical organoids expressed as a percentage of total colonies counted for each experimental condition from three independent experiments including the one shown in Fig 6B. **(B)** Bar graph depicts mean ± SEM proportion of CD44 immunoblot-derived signal of lysates of HCC38 cells transfected with the pU6 RNAi vector (vector control), or the plasmids CD44i-1, CD44i-2, alone or together (CD44i-1+2) from three independent experiments including the one shown in Fig 6D. **(C)** Bar graph depicts mean ± SEM proportion of spherical organoids expressed as a percentage of total colonies counted for untreated (-), 100 pM TGFβ or 400 μg/mL LMWHA-treated 6-day old three-dimensional organoids derived from HCC38 cells transfected with vector control or CD44i-1, CD44i-2, individually or in combination from three independent experiments including the one shown in Fig 6E. **(D)** Bar graph depicts mean ± SEM proportion of spherical organoids expressed as a percentage of total colonies counted for vector control or CD44/FLAG expressing-6 day-old HCC38 cell-derived organoids grown in complete growth medium without (-) or with 100 pM TGFβ or 400 μg/mL LMWHA, alone or with 100 μg/mL rhPRG4, from three independent experiments including the one shown in Fig 6G. Significant difference, ANOVA: *P ≤ 0.05, **P ≤ 0.01, ***P ≤ 0.001.
(TIF)

S4 Fig rhPRG4 reverses TGFβ-induced CD44-immunofluorescent derived signal in 3D-MDA-MB-231 cell derived organoids. Representative CD44 (Rat anti-CD44/anti-rat Alexa 647, red), and nuclei (Hoechst, blue) fluorescence microscopy images of fixed 8 day-old MDA-MB-231 cells-derived organoid that were incubated in complete growth medium without or with 100 pM TGFβ, alone or together with 100 μg/mL rhPRG4. The data are from an experiment that was repeated two times with similar outcomes. Scale bar indicates 50 μm.
(TIF)

S1 Appendix Raw data and analyses relating to the results shown in Figs 1–5, 7, 8 and S1–S3. Ten excel files, with each containing multiple sheets showing raw data and their analysis to determine the mean, standard deviation (SD) and standard error of mean (SEM) used to generate graphs illustrated in Figs 1–5, 7, 8 and **S1**–**S3**.
(ZIP)
