# Supplementary figures and images for: Recombinant human PRG4 (rhPRG4) suppresses breast cancer cell invasion by inhibiting TGFβ-Hyaluronan-CD44 signalling pathway
Source: PLoS One. 2019 Jul 30;14(7):e0219697. doi: 10.1371/journal.pone.0219697 (PMC6667139; doi:10.1371/journal.pone.0219697)

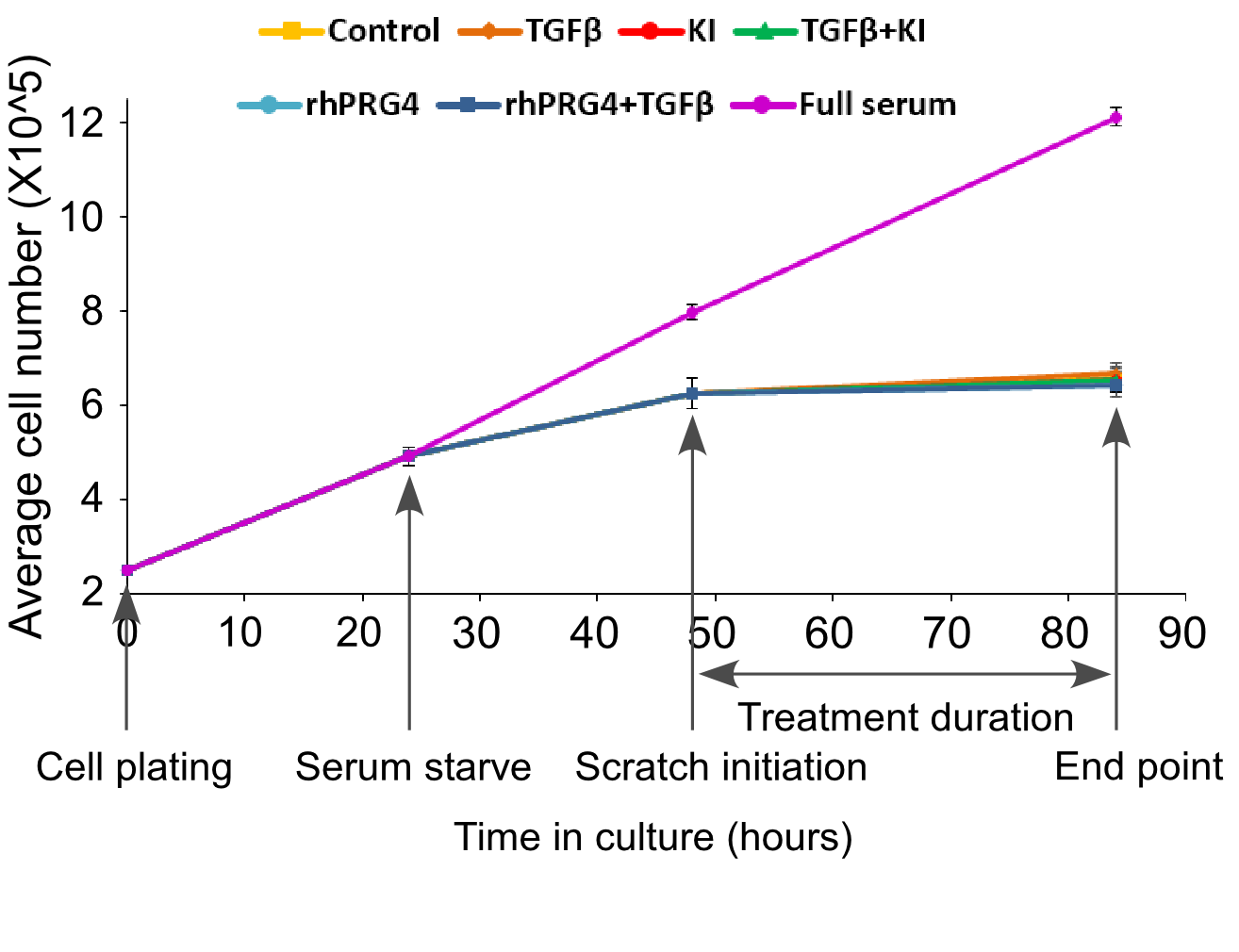

Supplement: S1 Fig — 2.5x10^5 MDA-MB-231 cells in 10% FBS-contaning growth medium were seeded per well of a 12-well tissue culture plate (0h). At 24h, medium was replaced with fresh DMEM with 10% FBS (full serum) or 0.2% serum (serum starve/low serum), followed by introducing a scratch per well (in all the wells) 24h later (or 48h post seeding). Cells were incubuated with regular or low serum medium, with and without TGFβ, KI or rhPRG4 in different combinations and left for another 36h post-scrach (or 84h post cell seeding). For each experiment, cell counts were obtained at the time points depicted in the graph, and average cell number ± SEM at each condition or timepoint from 3 independent experiments is plotted on y-axis versus time point on the x-axis. (TIF) [file pone.0219697.s001.tif]

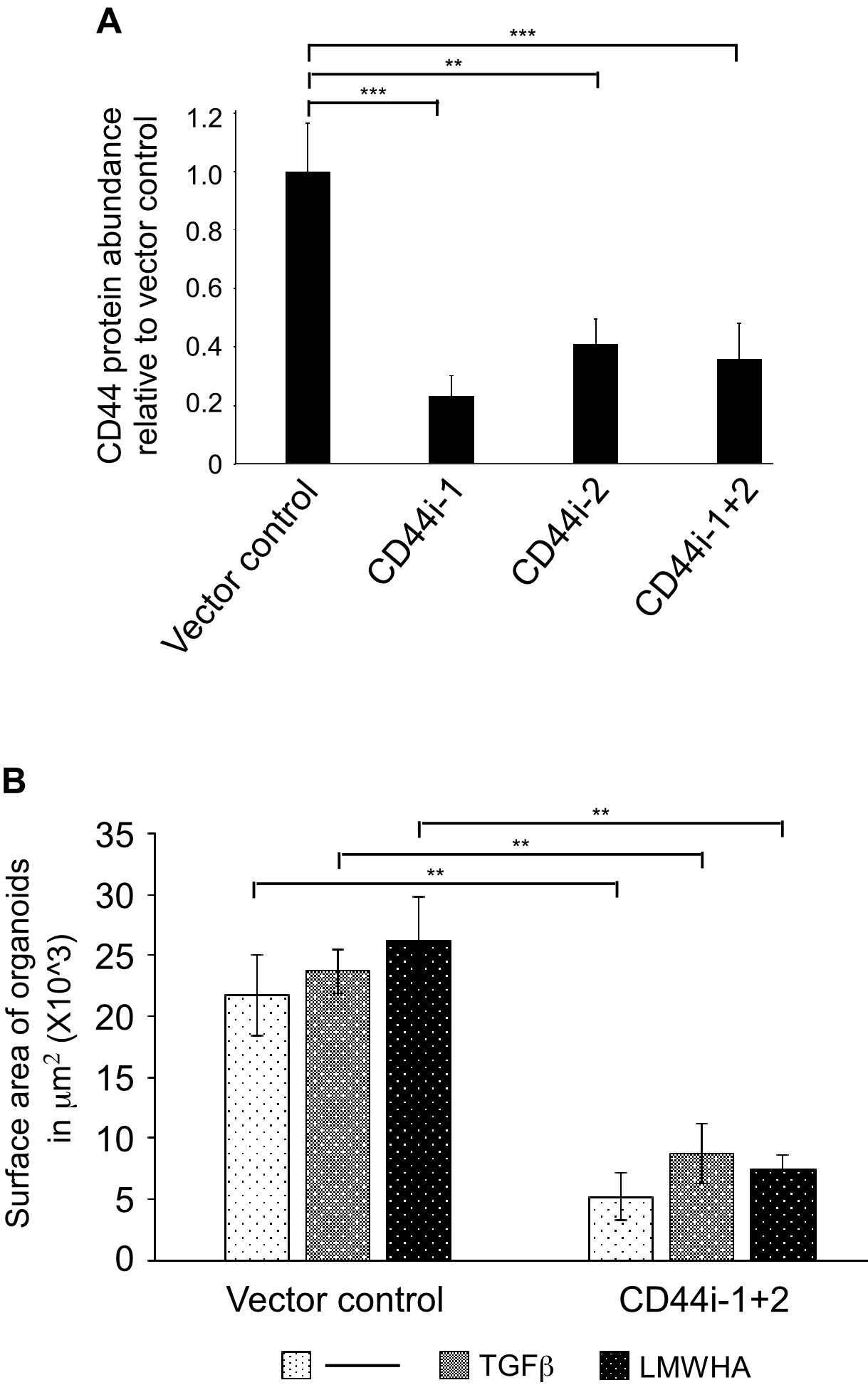

Supplement: S2 Fig — (A) Bar graph depicts mean ± SEM proportion of CD44 immunoblot-derived signals of lysates of MDA-MB-231 cells transfected with the pU6 RNAi vector (vector control), CD44 shRNA expressing plasmid CD44i-1 or CD44i-2, or in combination (CD44i-1+2), from experiments that were repeated three independent times including the one shown in Fig 5A. (B) Bar graph depicts mean ± SEM proportion of surface area of organoids derived from MDA-MB-231 cells transfected with the pU6 RNAi vector (vector control), or a combination of the CD44i-1and CD44i-2 plasmids (CD44i-1+2), before subjecting to 3D culturing, and leaving untreated (-) or incubating with 100 pM TGFβ or 400μg/mL of LMWHA, from experiments that were repeated three independent times including the one shown in Fig 5C. (TIF) [file pone.0219697.s002.tif]

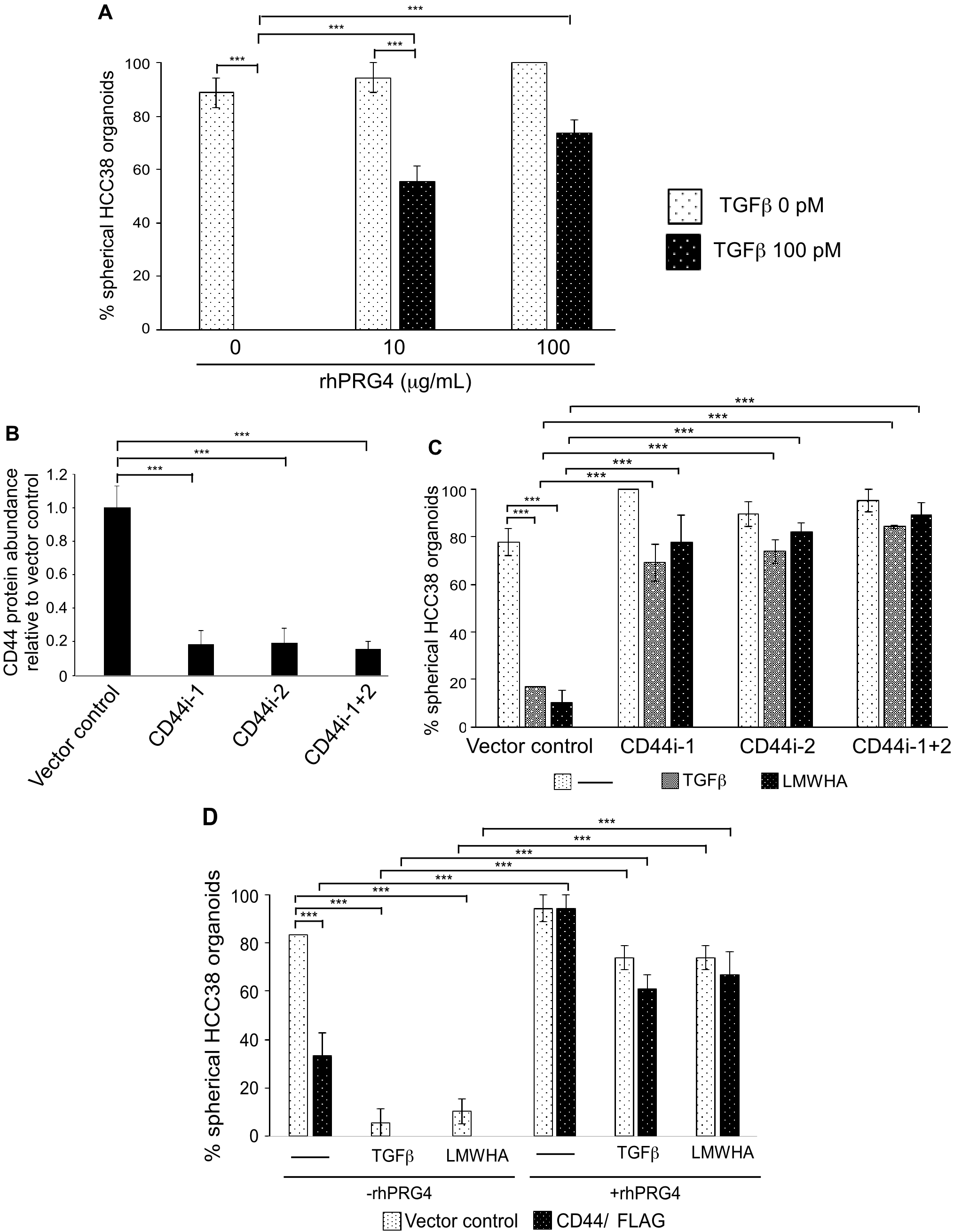

Supplement: S3 Fig — (A) Bar graph depicts mean ± SEM proportion of spherical organoids expressed as a percentage of total colonies counted for each experimental condition from three independent experiments including the one shown in Fig 6B. (B) Bar graph depicts mean ± SEM proportion of CD44 immunoblot-derived signal of lysates of HCC38 cells transfected with the pU6 RNAi vector (vector control), or the plasmids CD44i-1, CD44i-2, alone or together (CD44i-1+2) from three independent experiments including the one shown in Fig 6D. (C) Bar graph depicts mean ± SEM proportion of spherical organoids expressed as a percentage of total colonies counted for untreated (-), 100 pM TGFβ or 400 μg/mL LMWHA-treated 6-day old three-dimensional organoids derived from HCC38 cells transfected with vector control or CD44i-1, CD44i-2, individually or in combination from three independent experiments including the one shown in Fig 6E. (D) Bar graph depicts mean ± SEM proportion of spherical organoids expressed as a percentage of total colonies counted for vector control or CD44/FLAG expressing-6 day-old HCC38 cell-derived organoids grown in complete growth medium without (-) or with 100 pM TGFβ or 400 μg/mL LMWHA, alone or with 100 μg/mL rhPRG4, from three independent experiments including the one shown in Fig 6G. Significant difference, ANOVA: *P ≤ 0.05, **P ≤ 0.01, ***P ≤ 0.001. (TIF) [file pone.0219697.s003.tif]

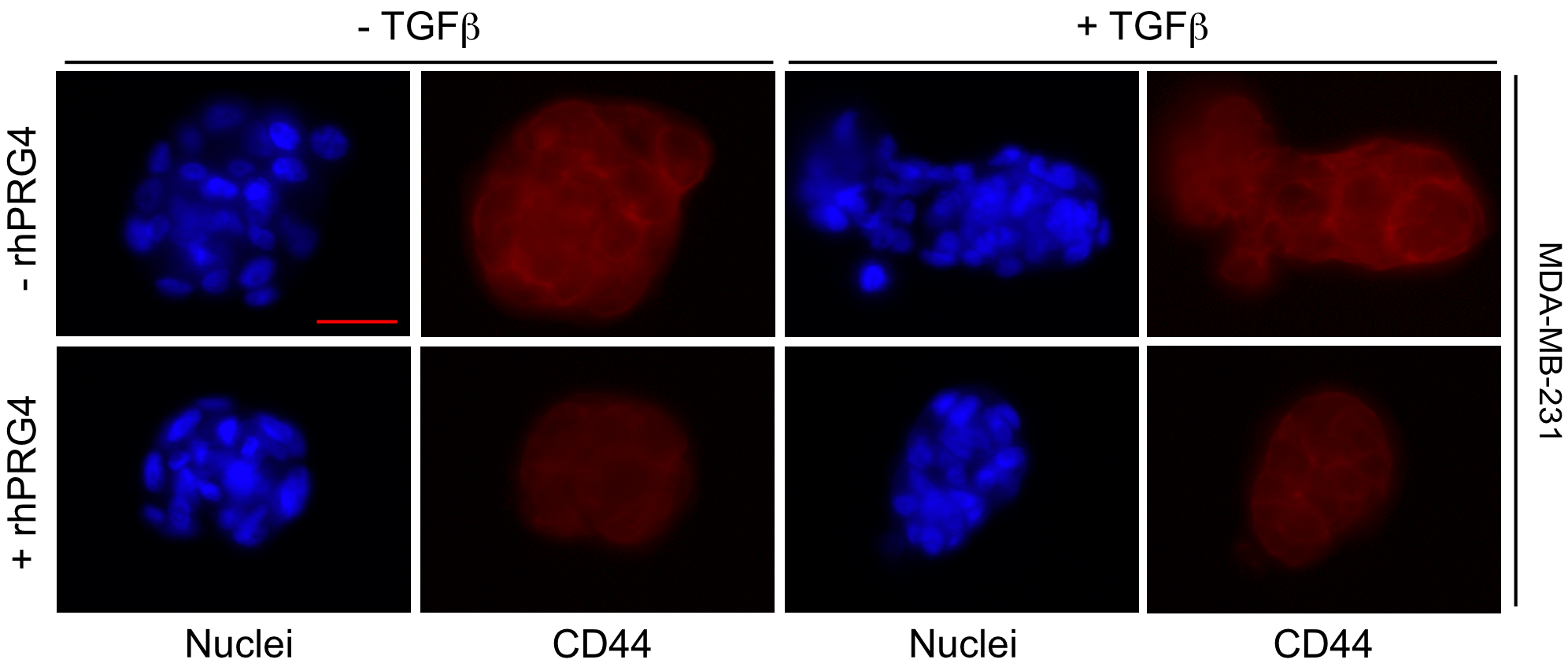

Supplement: S4 Fig — Representative CD44 (Rat anti-CD44/anti-rat Alexa 647, red), and nuclei (Hoechst, blue) fluorescence microscopy images of fixed 8 day-old MDA-MB-231 cells-derived organoid that were incubated in complete growth medium without or with 100 pM TGFβ, alone or together with 100 μg/mL rhPRG4. The data are from an experiment that was repeated two times with similar outcomes. Scale bar indicates 50 μm. (TIF) [file pone.0219697.s004.tif]
